# TGF‐β3 mediates mitochondrial dynamics through the p‐Smad3/AMPK pathway

**DOI:** 10.1111/cpr.13579

**Published:** 2023-11-27

**Authors:** Xinmei Du, Mengmeng Duan, Shiyi Kan, Yueyi Yang, Siqun Xu, Jieya Wei, Jiazhou Li, Hao Chen, Xuedong Zhou, Jing Xie

**Affiliations:** ^1^ State Key Laboratory of Oral Diseases, National Center for Stomatology, National Clinical Research Center for Oral Diseases, West China Hospital of Stomatology Sichuan University Chengdu Sichuan China

## Abstract

It is well recognized that mitochondrial dynamics plays a vital role in cartilage physiology. Any perturbation in mitochondrial dynamics could cause disorders in cartilage metabolism and even lead to the occurrence of cartilage diseases such as osteoarthritis (OA). TGF‐β3, as an important growth factor that appears in the joints of OA disease, shows its great potential in chondrocyte growth and metabolism. Nevertheless, the role of TGF‐β3 on mitochondrial dynamics is still not well understood. Here we aimed to investigate the effect of TGF‐β3 on mitochondrial dynamics of chondrocytes and reveal its underlying bio‐mechanism. By using transmission electron microscopy (TEM) for the number and morphology of mitochondria, western blotting for the protein expressions, immunofluorescence for the cytoplasmic distributions of proteins, and RNA sequencing for the transcriptome changes related to mitochondrial dynamics. We found that TGF‐β3 could increase the number of mitochondria in chondrocytes. TGF‐β3‐enhanced mitochondrial number was via promoting the mitochondrial fission. The mitochondrial fission induced by TGF‐β3 was mediated by AMPK signaling. TGF‐β3 activated canonical p‐Smad3 signaling and resultantly mediated AMPK‐induced mitochondrial fission. Taken together, these results elucidate an understanding of the role of TGF‐β3 on mitochondrial dynamics in chondrocytes and provide potential cues for therapeutic strategies in cartilage injury and OA disease in terms of energy metabolism.

## Graphical abstract

TGF‐β3 activates p‐Smad3 signalling and triggers the signalling cascade of AMPKα pathway to mediate mitochondrial dynamics. TGF‐β3 mediates chondrocytes to tilt towards the direction of mitochondrial fission and produces more mitochondrial fragments.

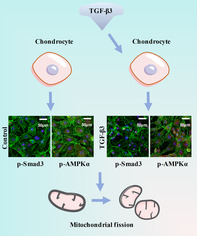

## 1 INTRODUCTION

Joint surfaces are coated with articular cartilage, which is a remarkably durable and smooth tissue, and offers exceptional resistance against mechanical strain.^1^ The extracellular matrix (ECM) of hyaline cartilage, which constitutes approximately 98% of the cartilage volume, primarily consists of hyaluronic acid, proteoglycans and collagen type II.^2^ Articular cartilage is populated exclusively by specialized chondrocytes. Under the co‐regulation of various cytokines, chondrocytes maintain the physiological and pathological activity of cartilage through balancing the anabolic and catabolic processes of ECM components.^3^, ^4^ However, due to the absence of nerves, blood vessels and lymphatic vessels, cartilage has limited regenerative capabilities following injury, making individuals susceptible to degenerative joint disorders such as osteoarthritis (OA).^5^, ^6^ OA, an age‐related degenerative joint condition, is characterized by the deterioration of articular cartilage, subchondral sclerosis, restricted joint mobility and discomfort.^7^ The aetiology of OA remains uncertain. Throughout the pathological progression of OA, various factors stimulate chondrocyte catabolism and disrupt cartilage homeostasis.^8^, ^9^ In OA conditions, chondrocytes display heightened catabolism and impaired mitochondrial function, leading to articular cartilage injury and matrix degradation.^10^, ^11^ Being the most prevalent rheumatic disease, the occurrence of OA increases with age and is more pronounced in individuals with metabolic syndrome or obesity.^12^, ^13^ Therefore, further elucidating the metabolic mechanisms of cartilage and the specific regulation of various growth factors could contribute to patient stratification and the development of novel therapeutics.

Mitochondria are essential organelles in chondrocytes that generate cellular energy in addition to glycolysis.^14^ Moreover, dysfunction of mitochondria has also been reported in the occurrence of OA diseases.^7^, ^15^ Some small molecules such as Urolithin A and melatonin have been shown to reduce cartilage damage and restore cartilage function in the progress of OA by improving mitochondrial function in chondrocytes.^16^, ^17^ Therefore, exploring the influence mechanisms of mitochondrial morphology and function changes in chondrocytes is conducive to the studies of new treatments for OA.

Mitochondrial dynamics is achieved through the dynamic balance of mitochondrial fusion and fission. Mitochondrial fusion proteins mainly include mitofusins (Mfn1/2), and optic atrophy 1 (Opa1).^18^ And dynamin‐related protein 1 (Drp1), and fission 1 protein (Fis1) are among the essential proteins responsible for mitochondrial fission processes.^19^ Recent reports have revealed significant advancements in mitochondrial dynamics through characterizing the key regulators involved in mitochondrial fusion and fission.^20^, ^21^ The Adenosine monophosphate‐activated protein kinase (AMPK) signalling plays significant roles in mitochondrial morphology.^22^ AMPK acts as a metabolic sensor, governing mitochondrial dynamics and maintaining energy balance.^23^

TGF‐β3 is a highly conservative and important growth factor of the TGF superfamily.^8^, ^24^, ^25^ TGF‐β3 can affect the chondrocyte behaviours to mediate cartilage homeostasis.^26^ In the process of OA, TGF‐β3 has dual effects: for healthy chondrocytes, it promotes cell vitality, however, for OA chondrocytes, it accelerates the progress of the disease.^27^ TGF‐β3 regulates cartilage though the Smad‐dependent pathway and the Smad‐independent pathway.^28^ Among them, the Smad‐dependent pathway is closely related to the metabolic activity of cartilage. TGF‐β3 also exerts anti‐inflammatory and pro‐inflammatory effects in different states of cartilage by mediating the Smad2/3 pathway and the Smad1/5/8 pathway, respectively.^29^ In recent years, TGF‐β3 had been used in therapeutic treatments of OA because it could promote the chondrogenic differentiation of mesenchymal stem cells and their migration to cartilage defects.^8^, ^9^

Limited studies have explored the effects of TGF‐β3 on mitochondria. It has been demonstrated that reduced TGF‐β3 release can contribute to obesity, insulin resistance, and fatty liver disease. This effect is linked to reduced mitochondrial respiration and anaerobic glycolysis in CD4^+^ T cells.^30^ Conversely, TGF‐β3 has been shown to down‐regulate mitochondrial respiration and enhance glycolysis in sertoli cells by suppressing Notch signalling activity.^31^, ^32^ However, the specific mechanisms by which TGF‐β3 regulates the morphology and dynamics of mitochondria in chondrocytes have not been comprehensively investigated.

In this study, we aimed to reveal the mitochondrial dynamics by characterizing mitochondrial fusion and fission in chondrocytes induced by TGF‐β3. We tried to show solid evidence to demonstrate the role of TGF‐β3 in mitochondrial dynamics and revealed its underlying bio‐mechanism in order to provide potential cues for cartilage repair.

## 2 MATERIALS AND METHODS

### 2.1 Cell culture

All tissue collection procedures were performed under the approval and guidance of Institutional Review Board (No. WCHSIRB‐D‐2021‐014) at the West China Hospital of Stomatology. As previously described,^33^ the study employed chondrocytes obtained from neonatal C57 mice, specifically aged between 3 and 5 days, for the investigation. The animals were euthanized in a humane manner, and the tissues were subjected to sterilization. Articular cartilage was isolated and fragmented into small sections. These sections were then incubated in 0.25% trypsin–EDTA solution for 30 min. The supernatant in the mixture is removed. The tissue underwent overnight digestion using 0.5% type II collagenase (No. C6885, Sigma‐Aldrich). The digestion reaction was terminated by adding configured 10% FBS DMEM (No. D6429, HyClone). Standard cell culture techniques were employed to cultivate the resulting cells.

### 2.2 Transmission electron microscopy

Chondrocytes were initially embedded in agarose tablets. Subsequently, they were treated with a 1% OsO_4_ solution at 4°C for 1 h. The sample was then subjected to dehydration, infiltration, and coating with resin, following the aforementioned methods.^13^, ^34^ To elaborate, the samples were incubated in different concentrations of ethanol and LX (25%, 50%, 75%, 95% and 100% ethanol at RT for 15 min); Ethanol: LX‐112 (3:1 and 1:3) at RT for 30 min; pure LX‐112 at RT for 60 min. Afterward, the sample was loaded into a pyramid tip mould (Ted Pella; 10585) and polymerized. Toluidine blue staining was performed to facilitate cell localization. Subsequently, ultra‐thin slices were cut, stained and placed on a grid. Finally, the sample was observed using an electron microscope (JEM‐1400FLASH, Japan). The desired image was captured using AMT‐600 image capture engine software and processed by photoshop software.

### 2.3 Mitochondrial staining of living cells

Chondrocytes were stained with Cell Navigator™ Mitochondrion Staining Kit (No. 22667, AAT Bioquest, CA, USA) in petri dishes specified for confocal laser microscopy for 30 min at 37°C as previously described.^13^ Then fixed the cells with 4% paraformaldehyde for 20 min and rinsed the cells with PBS three times. The samples were then imaged under a confocal laser scanning fluorescence microscope (FV3000, Olympus, Tokyo, Japan). The excitation/emission wavelengths were 540/590 nm (TRITC filter set). Nuclei were stained with DAPI (D9542, Sigma, USA) and the cytoskeleton was stained with FITC (A12379, Thermo, USA).

### 2.4 RNA sequencing and bioinformatics analysis

Chondrocytes were cultured onto a 6‐well plate and treated with TGF‐β3 (5 ng/mL) for 12 h. RNA sequencing between the control and TGF‐β3 treated chondrocytes was performed at Shanghai Lifegenes Biotechnology Co., Ltd. as previously described.^13^ Trizol reagent (Catalogue#15596026, Thermo Fisher Scientific, USA) was performed to extract RNA from chondrocytes. RNA integrity was evaluated at Illumina NovaSeq 6000 platform. HISAT2 v2.1.0 was used to match the reference genome and obtain raw data. GO enrichment analysis was performed using the DAVID database. The statistical significance of differentially expressed genes in KEGG database was detected with KOBAS v3.0 software. In the differential expression analysis, *p* < 0.05 and |FoldChange| ≥ 1.5 were the thresholds for significantly different expressions. Pheatmap was generated by an online R package.

### 2.5 Western blotting

The specific process followed the previous article.^35^ Briefly, proteins were isolated from cells by using RIPA lysis buffer and separated by 10% SDS‐polyacrylamide gel electrophoresis. Then they are transferred to PVDF membranes. Thereafter, the membranes were incubated with primary antibodies against β‐Actin (sc‐47778, Santa Cruz Biotechnology), Mfn1 (No. 509880, ZEN BIO), Mfn2 (No. 340604, ZEN BIO), Opa1 (No. 382025, ZEN BIO), phospho‐AMPKα1 (No. R26252, ZEN BIO), AMPKα1 (No. 380431, ZEN BIO), PGC1α (No. 381615, ZEN BIO), SIRT1 (No. R25721, ZEN BIO), Smad3 (#ab28379, Abcam), phospho‐Smad3 (No. R22919, ZEN BIO), Smad4 (#ab40759, Abcam), Drp1 (No. 221099, ZEN BIO) and Fis1 (No. R26001, ZEN BIO) at 4°C overnight, and the secondary antibodies against rabbit (sc‐2357, Santa Cruz Biotechnology) and mouse (sc‐516102, Santa Cruz Biotechnology) were incubated for 1 h at room temperature. Immobilon® Western kit (P90719, Millipore, Massachusetts, USA) was used to visualize the protein bands.

### 2.6 Immunofluorescence staining

Immunofluorescence staining was conducted following a previously described protocol.^36^ In brief, cells were incubated in a suitable petri dish. After 12 h, TGF‐β3 (5 ng/mL) (100‐36E, Pepro Tech) and/or SIS3 (CAS.1009104‐85‐1, Sigma‐Aldrich) were added to the experimental group. Incubation was continued for an additional 12 h. Then chondrocytes were sequentially washed three times with PBS. After being fixed with 4% paraformaldehyde for 10 min, the cells were rinsed with PBS three times. Then the cells were permeabilized with 0.25% Triton X‐100 for 10 min. Subsequently, the samples were incubated with specific primary antibodies. The primary antibodies used included anti‐rabbit Mfn1 (1:200, No. 509880, ZEN BIO), anti‐rabbit Mfn2 (1:200, No. 340604, ZEN BIO), anti‐rabbit Opa1 (1:200, No. 382025, ZEN BIO), anti‐rabbit phospho‐AMPKα1 (1:200, No. R26252, ZEN BIO), anti‐rabbit AMPKα1 (1:200, No. 380431, ZEN BIO), anti‐rabbit PGC1α (1:200, No. 381615, ZEN BIO), anti‐rabbit SIRT1 (1:200, No. R25721, ZEN BIO), anti‐rabbit Smad3 (1:200, #ab28379, Abcam), anti‐rabbit phospho‐Smad3 (1:200, No. R22919, ZEN BIO), anti‐rabbit Smad4 (1:200, #ab40759, Abcam), anti‐mouse Drp1 (1:200, No. 221099, ZEN BIO) and anti‐rabbit Fis1 (1:200, No. R26001, ZEN BIO). Each primary antibody was diluted at 1:1000. Then the cells were stained with secondary antibody using Alexa Fluor® 647 (#ab150075, Abcam) for 2 h in the dark. Then the cytoskeleton was stained with FITC using Alexa Fluor® 488 (#ab150077, Abcam). After 10 min fixation with DAPI‐containing mounting fluid (D9542, Sigma Aldrich), the cells were imaged via a confocal laser scanning microscope FV3000, which enabled visualization and analysis of the localization and distribution of specific proteins within cells.

### 2.7 ATP assay

ATP detection was done as described earlier.^37^ In short, we used the enhanced ATP assay kit (No. S0027, Beyotime) to detect the ATP products according to the instructions. The cells were first lysed with ATP lysis buffer. Then the sample was collected by centrifuged at 15,000*g* at 4°C for 10 min. After that, ATP test standard solution and ATP working solution would be prepared according to the instructions. The diluted ATP working solution was added to the sample and mixed well. These operations took place in the dark. Finally, the ATP level was measured by using a luminometer (Bio Tek Instrument, USA).

### 2.8 Statistical analysis

All Data were reported as mean ± standard deviation (SD) of at least three independent experiments (*n* ≥ 3). The data were analysed by a two‐detailed Student *t*‐test. Differences were considered to be significant when *p*‐value was less than 0.05.

## 3 RESULTS

### 3.1 TGF‐β3 enhanced the number of mitochondria in chondrocytes

**FIGURE 1:**
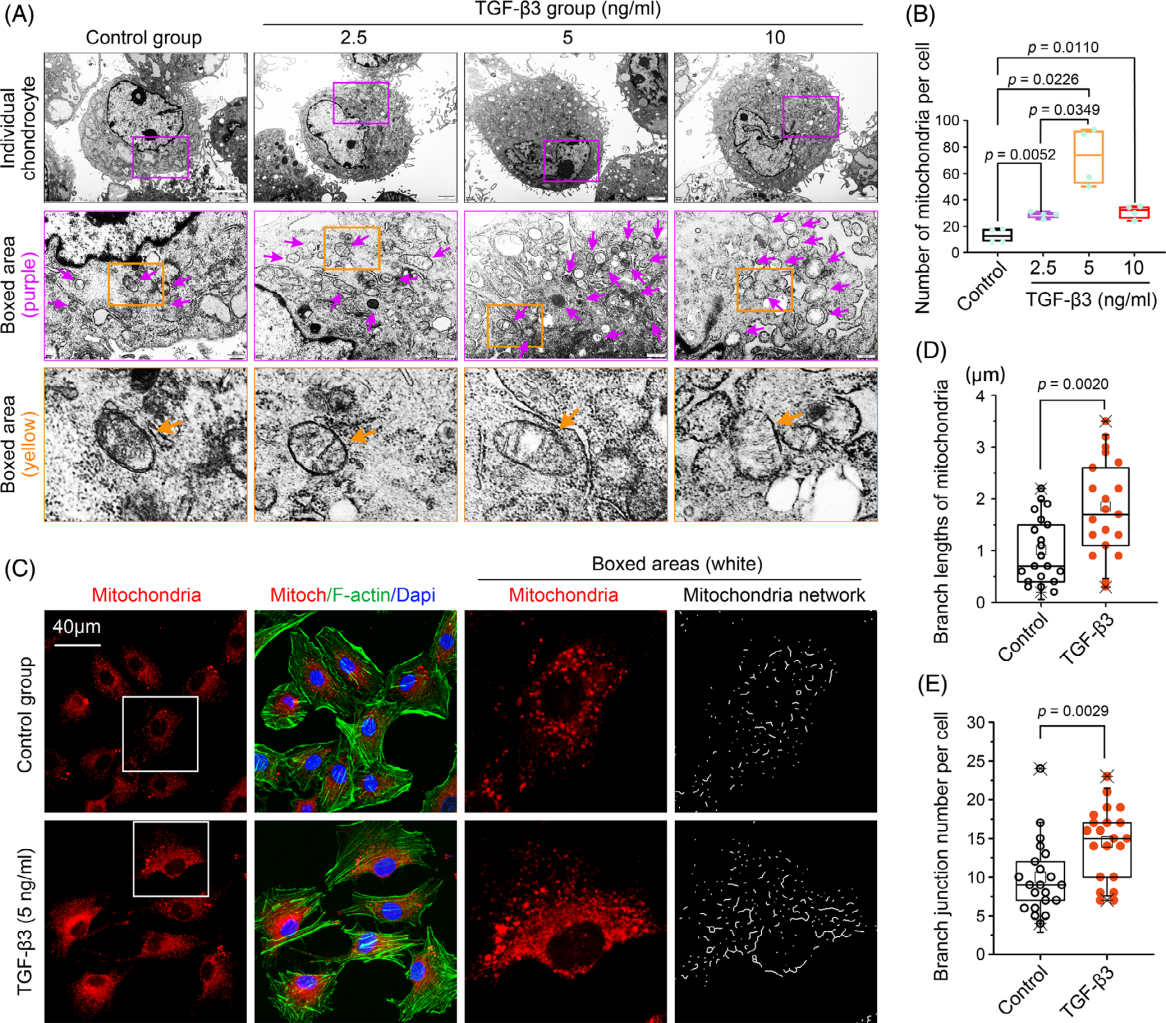
TGF‐β3 increased the number of mitochondria in chondrocytes. (A) Representative transmission electron microscopy images showed that TGF‐β3 up‐regulated the number of mitochondria in chondrocytes at different concentrations. The results were based on three independent experiments (*n* = 3). (B) Bar graphs showed the quantitative analysis of the number of mitochondria per chondrocyte shown in (A). The results were based on three independent experiments (*n* = 3). (C) Mitochondrial staining demonstrated the number of changes in mitochondria in chondrocytes induced by TGF‐β3 at 5 ng/mL. The results were based on three independent experiments (*n* = 3). Mitochondria, red; Cytoskeleton (F‐actin), green; Nucleus (Dapi), blue. (D) Quantitative analysis of branch lengths in mitochondria network in chondrocytes induced by TGF‐β3 at 5 ng/mL. The results were based on three independent experiments (*n* = 3). (E) Quantitative analysis of branch junctions in mitochondria network in chondrocytes induced by TGF‐β3 at 5 ng/mL. The results were based on three independent experiments (*n* = 3). The data in (B, D, E) were shown as box (from 25, 50 to 75%) and whisker (standard deviation, SD). Student's *t*‐test was applied to determine the significant differences in (B, D, E).

To detect the influence of TGF‐β3 on mitochondrial dynamics in chondrocytes, we first used the transmission electron microscopy (TEM) and found an increase in mitochondria number following 12 h treatment with TGF‐β3 (2.5, 5 and 10 ng/mL) relative to the control group (Figure 1A, purple and yellow arrows indicated the individual mitochondria). Quantitative analysis confirmed the increase of mitochondria number in chondrocytes induced by TGF‐β3 at different concentrations (Figure 1B). Importantly, we noticed that the effect of TGF‐β3 at 5 ng/mL on mitochondria number was much more prominent relative to those at other concentrations. From the perspective of ATP generation, we also found that TGF‐β3 could increase the ATP products in chondrocytes at different concentrations (Figure S1), especially at 5 ng/mL. We next used mito‐tracker to label mitochondria in living chondrocytes (Figure 1C), and found that TGF‐β3 at 5 ng/mL could greatly enhance the mitochondria number, and moreover, these enhanced mitochondria formed an augmented mitochondria network (Right boxed areas indicated by ImageJ). Analysis of mitochondria network showed that mitochondria‐formed branch lengths (Figure 1D) and branch junctions (Figure 1E) were significantly increased in chondrocytes induced by TGF‐β3 at 5 ng/mL. Collectively, these results established a preliminary correction between TGF‐β3 and mitochondrial dynamics.

### 3.2 TGF‐β3 influences mitochondrial dynamics by increasing mitochondrial fission and decreasing mitochondrial fusion

**FIGURE 2:**
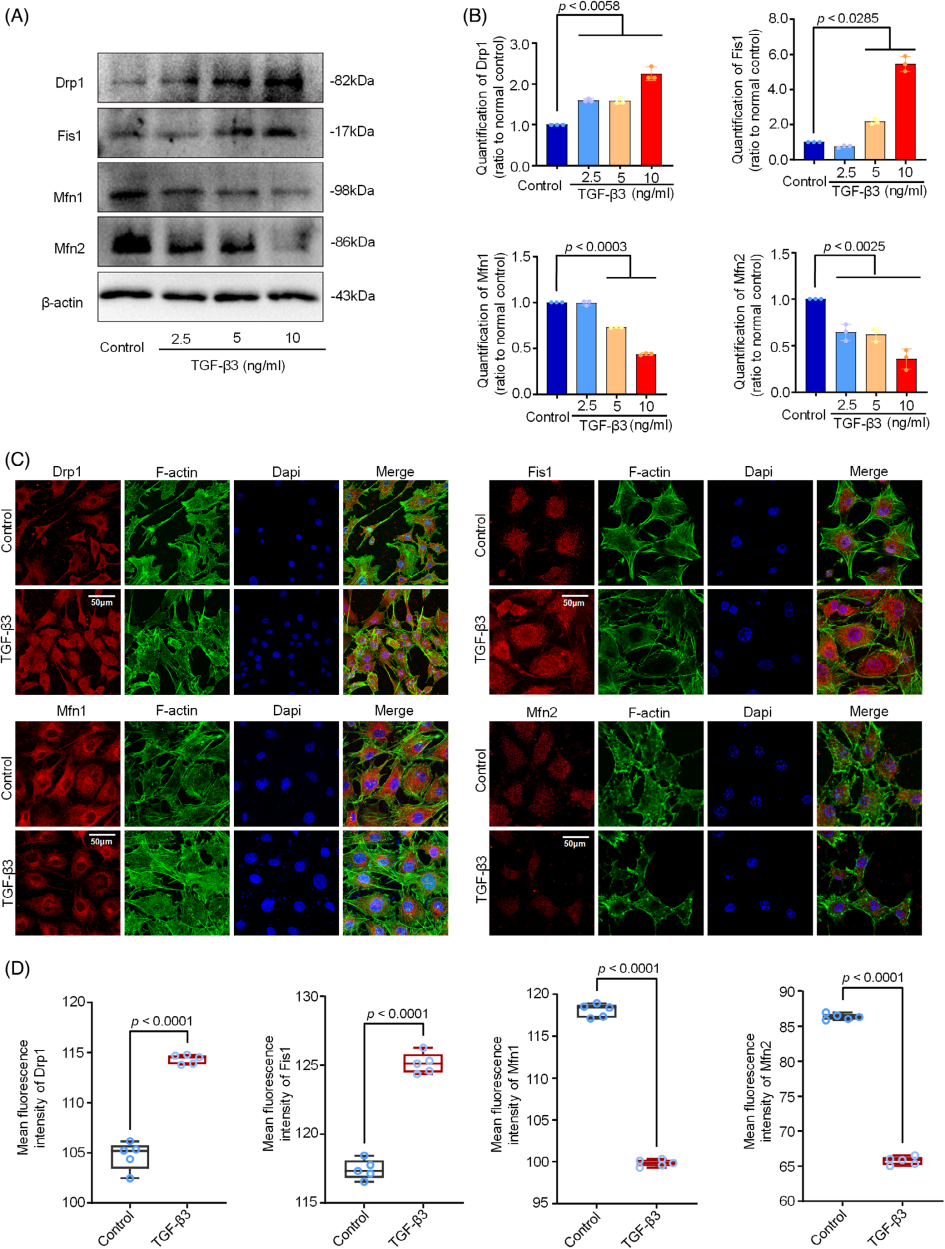
TGF‐β3 induced the expression of mitochondrial fusion and fission‐related proteins in chondrocytes. (A) Representative western blotting images showed the expressions of Drp1, Fis1, Mfn1 and Mfn2 in chondrocytes induced by TGF‐β3 with different concentrations (2.5, 5 or 10 ng/mL) for 12 h. The results were based on three independent experiments (*n* = 3). (B) Bar graphs showed the quantification of mitochondrial fusion and fission‐related proteins shown in (A). The results were based on three independent experiments (*n* = 3). (C) Representative immunofluorescent images showed the changes of mitochondrial fusion and fission‐related proteins in chondrocytes induced by TGF‐β3 at 5 ng/mL. The results were based on three independent experiments (*n* = 3). Mitochondrial fusion or fission proteins, red; Cytoskeleton (F‐actin), green; Nucleus (Dapi), blue. (D) Quantitative analysis indicated the expressions of mitochondrial fusion and fission‐related proteins in chondrocytes induced by TGF‐β3 at 5 ng/mL. The results were based on three independent experiments (*n* = 3). The data in (B) were presented as the means ± SD. The data in (D) were shown as box (from 25, 50 to 75%) and whisker (SD). Student's *t*‐test is applied to determine the significant differences.

To evaluate the impact of TGF‐β3 on mitochondrial fusion and fission in chondrocytes, we investigated the protein markers, including Drp1 and Fis1 which are responsible for mitochondrial fission,^18^ and Mfn1/2 and Opa1 which are in charge of mitochondrial fusion.^19^ By western blotting (Figure 2A), we found the expressions of Drp1 and Fis1 were significantly enhanced after TGF‐β3 treatment for 24 h. In contrast, the expressions of Mfn1 and Mfn2 were significantly reduced in chondrocytes induced by TGF‐β3 at different concentrations. Quantitative analysis of Drp1, Fis1, Mfn1 and Mfn2 confirmed these results (Figure 2B). As for the expressions of Opa1, an inner mitochondrial membrane fusion protein,^38^ showed an unstable change in chondrocytes induced by TGF‐β3 for 24 h (Figure S2). We next investigated the cytoplasmic distribution changes of those protein markers by immunofluorescence (Figure 2C) and the results showed that the cytoplasmic expressions of Drp1 and Fis1 were increased while the expression of Mfn1 and Mfn2 were decreased as quantified in Figure 2D, which were consistent with the western blotting results.

**FIGURE 3:**
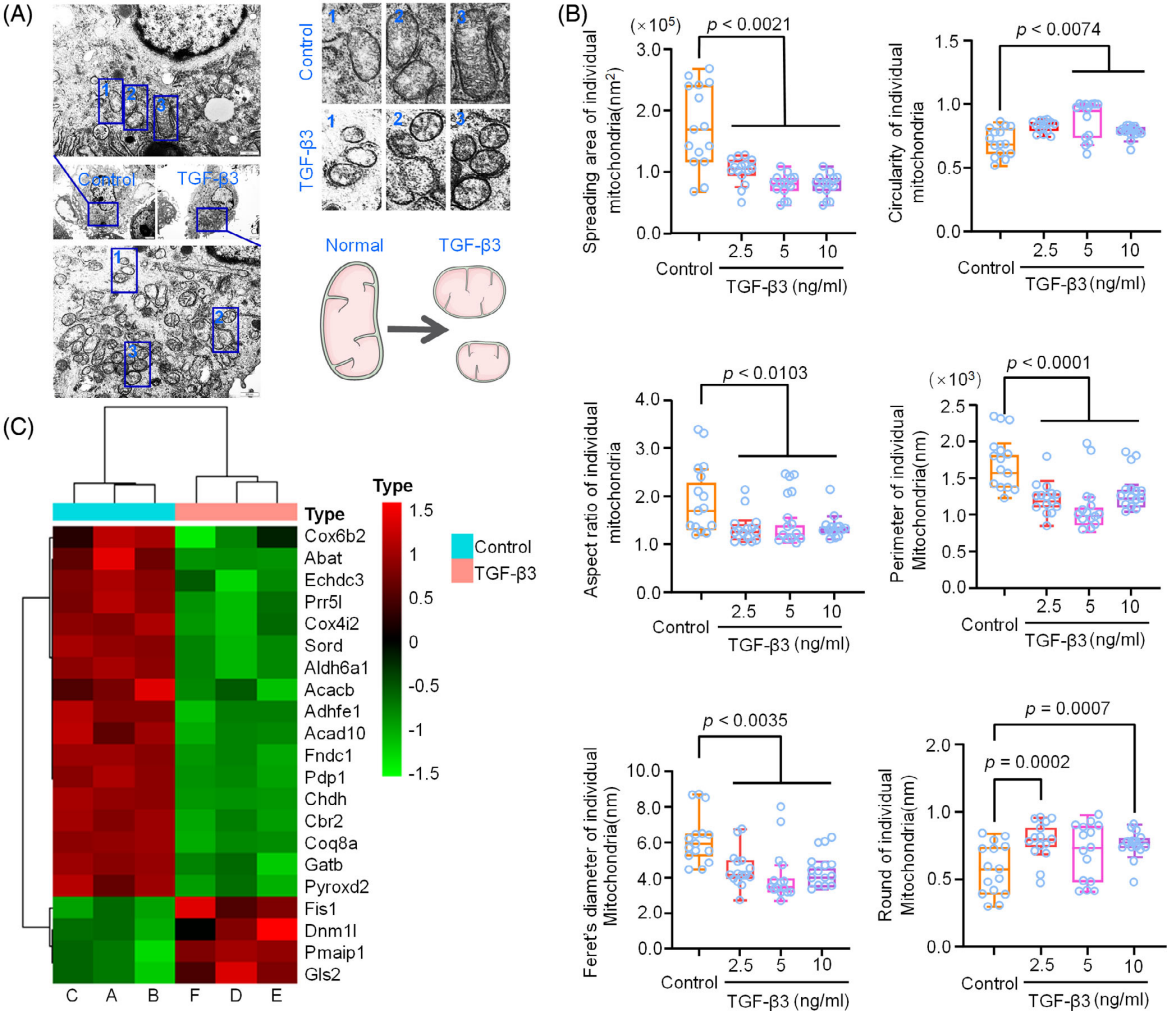
Characterization of mitochondria induced by TGF‐β3. (A) Representative transmission electron microscopy images showed the changes in mitochondrial fission in chondrocytes induced by TGF‐β3 at 5 ng/mL. The results were based on three independent experiments (*n* = 3). The schematic diagram in the lower right corner further illustrates mitochondrial fission induced by TGF‐β3. (B) Quantitative analysis based on ImageJ‐mitochondria network analysis showed the changes in the mitochondrial spreading area, circularity, aspect ratio, perimeter, Feret's diameter and round form of individual mitochondria in chondrocytes induced by TGF‐β3 at different concentrations (2.5, 5 and 10 ng/mL). The results were based on three independent experiments (*n* = 3). (C) RNA sequencing showing the changes in the 21 mitochondria‐related genes in chondrocytes induced by TGF‐β3 (5 ng/mL). Three independent pairs of samples, A and A′, B and B′, C and C′, were used to obtain the RNA sequencing data. The results were based on three independent experiments (*n* = 3). The data were presented as log2(FPKM+1). The data in (B) were shown as box (from 25, 50 to 75%) and whisker (standard deviation, SD). Student's *t*‐test was applied to determine the significant differences.

Based on the above results that TGF‐β3‐mediated mitochondrial dynamics led to a favourable fission, we further explored the mitochondrial fission by characterizing mitochondrial number and morphology of individual chondrocytes by TEM (Figure 3A). The results indicated that mitochondria in individual chondrocytes induced by TGF‐β3 (5 ng/mL) exhibit a larger number, but these increased numbers of mitochondria showed a smaller size and a rounder morphology compared to the normal ones (control group). We further analysed the morphological parameters of mitochondria in chondrocytes induced by TGF‐β3 at different concentrations by ImageJ (Figure 3B). From the quantitative analysis of the spreading area, mitochondrial aspect ratio, perimeter and Feret's diameter of the individual mitochondria, we found that these parameters in chondrocytes induced by TGF‐β3 were all decreased. Meanwhile, from the analysis of the circularity and round form of these individual mitochondria, we found the parameters were increased. Moreover, based on RNA sequencing, we showed that the expressions of the genes that regulated mitochondrial function were generally impaired in chondrocytes induced by TGF‐β3 (Figure 3C). Taken together, these data indicated TGF‐β3 promoted the mitochondrial fission and generated much smaller mitochondria in chondrocytes, which would contribute to potential mitochondrial fragmentation.

### 3.3 TGF‐β3 activates AMPK signalling to regulate mitochondrial dynamics

**FIGURE 4:**
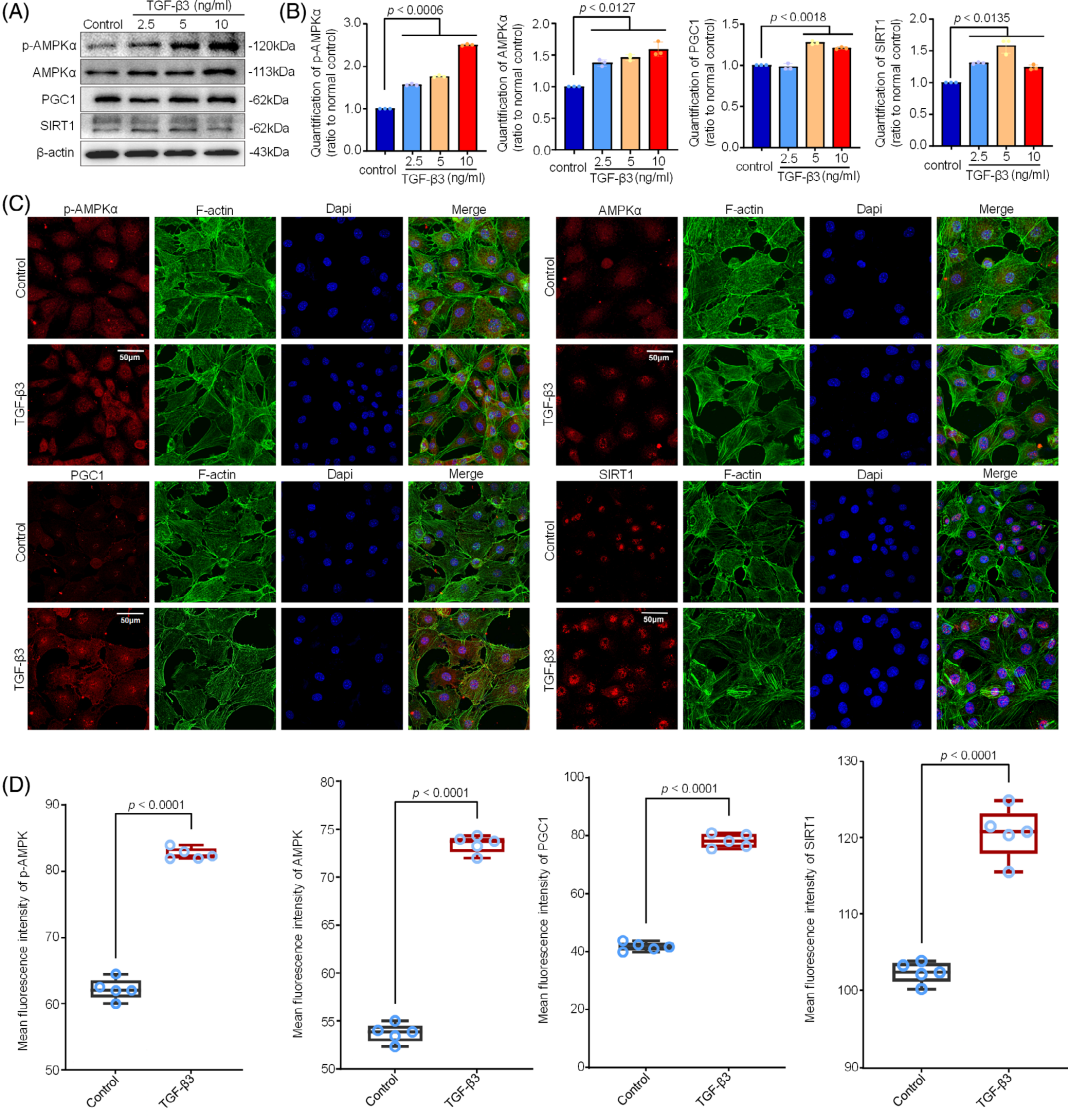
TGF‐β3 activated AMPKα signalling in chondrocytes. (A) Representative western blotting images showed the expressions of p‐AMPKα, AMPKα, PGC1 and SIRT1 in chondrocytes induced by TGF‐β3 at different concentrations (2.5, 5 and 10 ng/mL) for 12 h. The results were based on three independent experiments (*n* = 3). (B) Bar graphs showed the quantification of the expressions of p‐AMPKα, AMPKα, PGC1 and SIRT1 in chondrocytes induced by TGF‐β3 at different concentrations shown in (A). The results were based on three independent experiments (*n* = 3). (C) Immunofluorescence staining showed the cytoplasmic expression and distribution of p‐AMPKα, AMPKα, SIRT1, and PGC1 in chondrocytes induced by TGF‐β3 at 5 ng/mL. The results were based on three independent experiments (*n* = 3). *p*‐AMPKα, AMPKα, SIRT1, and PGC1, red; Cytoskeleton (F‐actin), green; Nucleus (Dapi), blue. (D) Quantitative analysis indicated the expressions of AMPKs in chondrocytes induced by TGF‐β3 at 5 ng/mL. The results were based on three independent experiments (*n* = 3). The data in (B) were presented as the means ± SD. The data in (D) were shown as box (from 25, 50 to 75%) and whisker (SD). Student's *t*‐test was applied to determine the significant differences.

It is well recognized that AMPK signalling is the direct upstream in mediating mitochondrial fission and fusion via AMPKα/PGC‐1α/SIRT1.^13^, ^39^ Thus, we explored the change of AMPK signalling in chondrocytes induced by TGF‐β3. We found that the expressions of total and phosphorylated AMPKα were increased in chondrocytes induced by TGF‐β3 at different concentrations (Figure 4A). Moreover, the protein expressions of PGC‐1α and SIRT1 were also increased in chondrocytes induced by TGF‐β3 at different concentrations. These increases in AMPK signalling‐related proteins were further confirmed by quantitative analysis (Figure 4B). We next explored the cytoplasmic distribution changes of these proteins. By using immunofluorescence, we showed the expressions of these proteins in chondrocytes and found that TGF‐β3 at 5 ng/mL could enhance the cytoplasmic expressions of these proteins (Figure 4C). Quantitative analysis further confirmed the results (Figure 4D).

### 3.4 TGF‐β3 activates p‐Smad3 signalling to regulate mitochondrial dynamics

**FIGURE 5:**
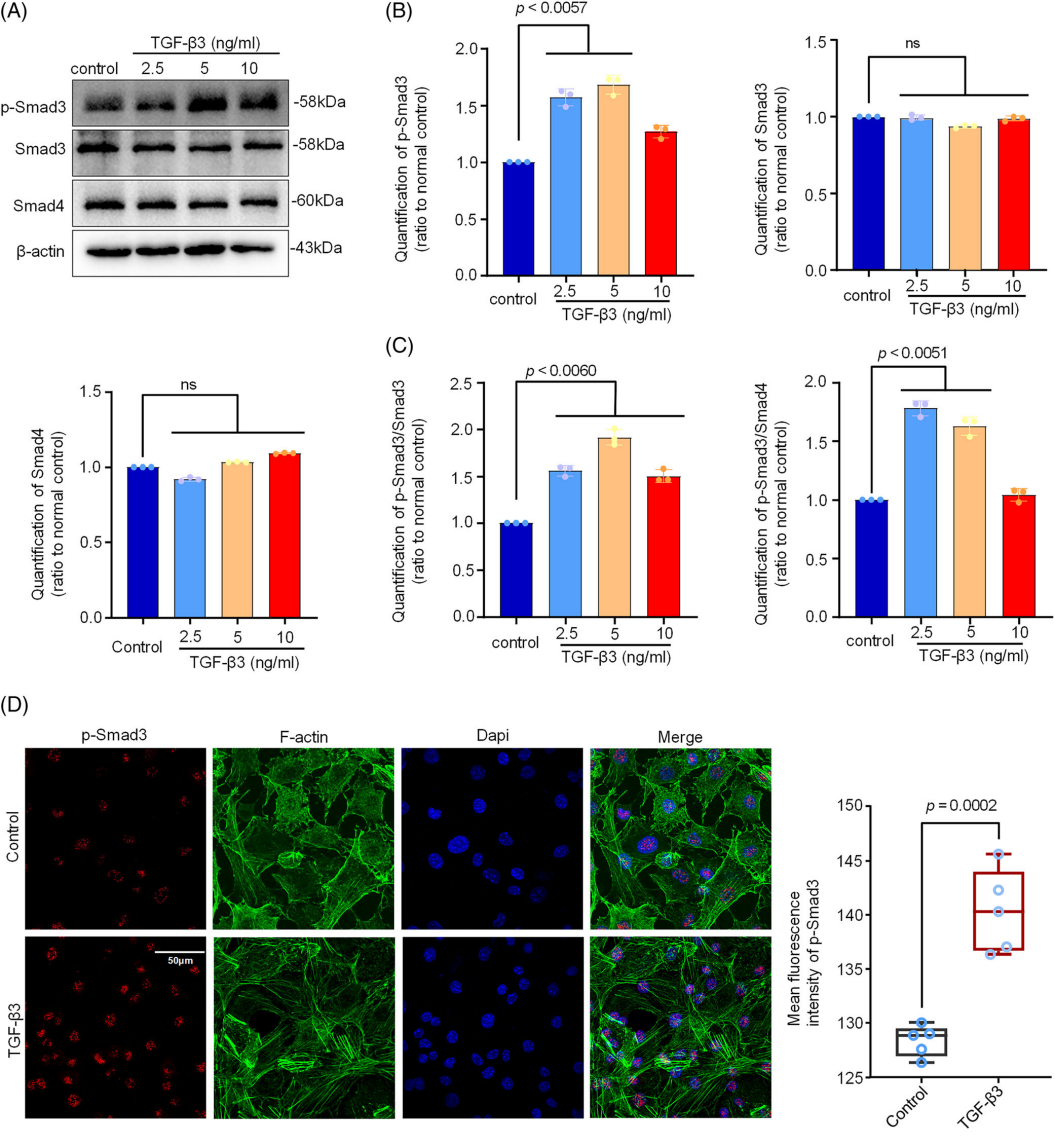
TGF‐β3 activated canonical p‐Smad3 signalling in chondrocytes. (A) Representative western blotting images showed the protein expression of p‐Smad3, Smad3 and Smad4 in chondrocytes induced by TGF‐β3 at different concentrations (2.5, 5, and 10 ng/mL).The results were based on three independent experiments (*n* = 3). (B) Bar graphs showed the quantification of p‐Smad3, Smad3 and Smad4 in chondrocytes induced by TGF‐β3 at different concentrations. The results were based on three independent experiments (*n* = 3). (C) Quantification further showed the ratio changes of p‐Smad3/Smad3 and p‐Smad3/Smad4 in chondrocytes induced by TGF‐β3 at different concentrations. The results were based on three independent experiments (*n* = 3). (D) Immunofluorescence staining showed the expression and distribution of p‐Smad3 in chondrocytes induced by TGF‐β3 at 5 ng/mL. The results were based on three independent experiments (*n* = 3). p‐Smad3 proteins, red; Cytoskeleton (F‐actin), green; Nucleus (Dapi), blue. The data in (B, C) were presented as the means ± SD. The data in (D) were shown as box (from 25, 50 to 75%) and whisker (SD). Student's *t*‐test was applied to determine the significant differences.

The Smad3 signalling has been considered to be a canonical signalling in the regulation of cartilage metabolism mediated by TGF‐βs.^29^, ^40^ To further explore the cytoplasmic signalling in mediating AMPK‐regulated mitochondrial dynamics in chondrocytes induced by TGF‐β3, we detected the change of Smad3/Smad4 complex in chondrocytes induced by TGF‐β3. By western blotting, we found that the expression of p‐Smad3 was greatly enhanced in chondrocytes although the total expressions of Smad3 and Smad4 were unchanged (Figure 5A). Quantitative analysis of these proteins confirmed these changes (Figure 5B). We next analysed the ratio of p‐Smad versus Smad3 and Smad4, and the results clearly illustrate the increased ratio in phosphorylation of Smad3 (Figure 5C). From immunofluorescence, we further observed the accumulation of p‐Smad3 in the nuclear region of chondrocytes induced by TGF‐β3 at 5 ng/mL was higher than that of normal chondrocytes (Figure 5D). Collectively, these results indicated TGF‐β3 activated the p‐Smad3 signalling in chondrocytes.

### 3.5 TGF‐β3 mediates mitochondrial dynamics in chondrocytes via p‐Smad3/AMPK signalling

**FIGURE 6:**
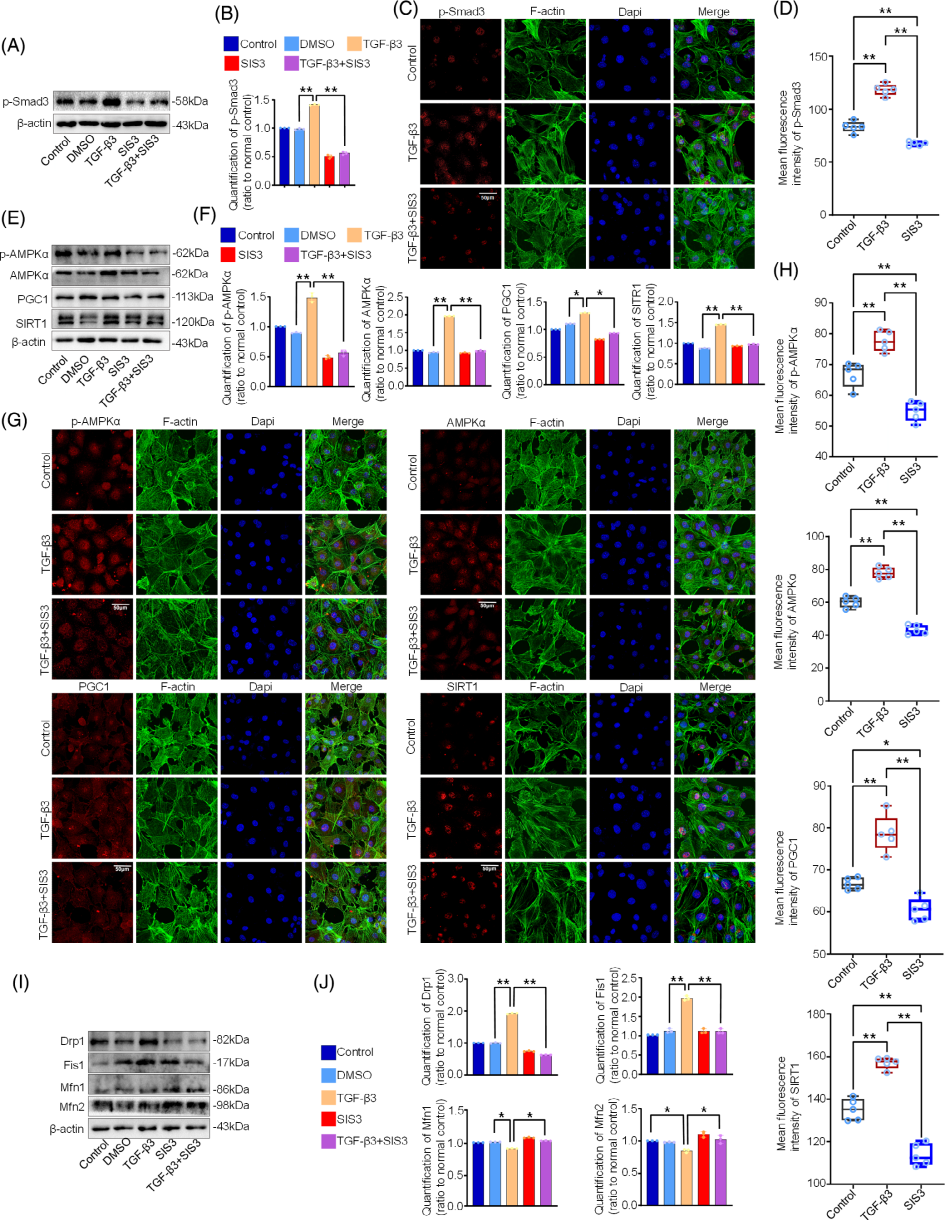
TGF‐β3 activated p‐Smad3/AMPK signalling to mediate mitochondria dynamics. (A) Representative western blotting images showed the expressions of p‐Smad3 in chondrocytes treated with SIS3, a specific inhibitor of p‐Smad signalling, in the presence of TGF‐β3 at 5 ng/mL. The results were based on three independent experiments (*n* = 3). (B) Bar graphs showed the quantification of p‐Smad3 protein in (A). The results were based on three independent experiments (*n* = 3). (C) Immunofluorescence staining showed the effect of SIS3 on the expression of p‐Smad3 signalling in the presence of TGF‐β3 at 5 ng/mL. The results were based on three independent experiments (*n* = 3). (D) Quantitative analysis indicated the expressions of p‐Smad3 in chondrocytes induced by SIS3 in the presence of TGF‐β3 at 5 ng/mL. The results were based on three independent experiments (*n* = 3). (E) Representative western blotting images showed the expressions of p‐AMPK, AMPK‐α, PGC1 and SIRT1 in chondrocytes treated by SIS3 in the presence of TGF‐β3 at 5 ng/mL. The results were based on three independent experiments (*n* = 3). (F) Bar graphs showed the quantification of p‐AMPK, AMPK‐α, PGC1 and SIRT1 in chondrocytes treated by SIS3 in the presence of TGF‐β3 at 5 ng/mL. The results were based on three independent experiments (*n* = 3). (G) Immunofluorescence staining showed the expression and distribution of p‐AMPK, AMPK‐α, PGC1 and SIRT1 in chondrocytes treated with SIS3 in the presence of TGFβ3 at 5 ng/mL. The results were based on three independent experiments (*n* = 3). (H) Quantitative analysis indicated the expressions of AMPKs in chondrocytes induced by SIS3 in the presence of TGF‐β3 at 5 ng/mL. The results were based on three independent experiments (*n* = 3). (I) Representative western blotting images showed the expressions of Drp1, Fis1, Mfn1 and Mfn2 in chondrocytes treated with SIS3 in the presence of TGF‐β3 at 5 ng/mL for 12 h. The results were based on three independent experiments (*n* = 3). (J) Bar graphs showed the quantification of the expressions of Drp1, Fis1, Mfn1 and Mfn2 in chondrocytes treated with SIS3 in the presence of TGF‐β3 at 5 ng/mL. The results were based on three independent experiments (*n* = 3). The data in (B, F, J) were presented as the means ± SD. The data in (H) were shown as box (from 25, 50 to 75%) and whisker (SD). Student's *t*‐test was applied to determine the significant differences. *p < 0.05; **p < 0.01.

To further confirm the role of p‐Smad3 signalling in TGF‐β3‐mediated mitochondrial dynamics in chondrocytes, we applied SIS3, a specific Smad3 signalling inhibitor,^41^ to show the influence of Smad3 signalling on the regulations of AMPK and the protein candidates in mitochondrial dynamics. We first detected the effect of SIS3 on the expression of p‐Smad3 (Figure 6A). The result showed that SIS3 could effectively reduce the expression of p‐Smad3 even in the presence of TGF‐β3 at 5 ng/mL. Quantitative analysis confirmed the change of this result (Figure 6B). Furthermore, by using immunofluorescence, we confirmed the protein decrease of p‐Smad3 induced by SIS3 in chondrocytes in the presence of TGF‐β3 at 5 ng/mL (Figure 6C,D). Next, we investigated the influence of p‐Smad3 signalling on the regulations of AMPK. We found that blockage of p‐Smad3 signalling decreased the expression of AMPK, p‐AMPK, PGC1 and SIRT1 (Figure 6E). These decreased expressions were further confirmed by quantitative analysis based on greyscale value (Figure 6F). We also used immunofluorescence to detect the cytoplasmic distributions of these proteins, that is, AMPK, p‐AMPK, SIRT1 and PGC1 (Figure 6G). The results of immunofluorescence staining showed the expressions of AMPK, p‐AMPK, SIRT1 and PGC1 were all decreased after blockage of p‐Smad3 signalling in the presence of TGF‐β3 at 5 ng/mL. Quantitative analysis of immunofluorescence further confirmed the results (Figure 6H). Finally, we examined the influence of p‐Smad3 signalling on the proteins that were in charge of mitochondrial dynamics. We found that the enhanced expressions of fission proteins, Drp1 and Fis1, in chondrocytes induced by TGF‐β3 were greatly reduced after treatment with SIS3 and the impaired expression of Mfn1 and Mfn2 induced by TGF‐β3 were restored to certain extent (Figure 6I,J). Taken together, these data indicated TGF‐β3 mediates mitochondrial fission of chondrocytes via p‐Smad3/AMPK signalling.

## 4 DISCUSSION

Mitochondria play a crucial role in the energy generation of chondrocytes and thus significantly influence the physiological processes of cartilage. Mitochondrial dysfunction contributes to the pathogenesis of OA.^13^, ^18^ TGF‐β3 plays an important role in the metabolism of chondrocytes, including chondrocyte proliferation, migration, differentiation and death.^42^ TGF‐β3, known for its involvement in chondrocyte metabolism, has emerged as a promising candidate for treating cartilage defects by promoting chondrogenesis in mesenchymal stromal cells through activation of the Smad‐dependent pathway.^21^, ^40^, ^43^ In the current study, we observed that chondrocytes showed up‐regulated mitochondrial fission and down‐regulated mitochondrial fusion after treatment with TGF‐β3 for 12 h. The number of mitochondria in chondrocytes transiently increased, accompanied by elevated ATP production. TGF‐β3 activates the AMPK to regulate mitochondrial dynamics and this regulation was controlled by cytoplasmic p‐Smad3 signalling. These results help to understand the role of TGF‐β3 in chondrcoyte behaviours, especially in mitochondrial dynamics.

The destruction of articular cartilage in the progress of OA is primarily attributed to the imbalance of energy metabolism in chondrocytes, leading to ATP and NADPH depletion.^8^ Existing biological therapies for OA have failed to effectively correct the metabolic imbalance in damaged and degenerative chondrocytes, resulting in poor clinical prognosis.^44^ Previous researches have established the significant involvement of TGF‐β3 in the physiological and pathological metabolic activities in chondrocytes.^4^, ^21^ However, there have been few studies to explore the specific mechanisms and regulatory effects of TGF‐β3 on mitochondria. In our current study, we observed a significant increase in mitochondrial quantity under the treatment of TGF‐β3, which was confirmed by TEM and immunofluorescence staining (Figures 1A,C and 3A). Mitochondria are important sites for ATP synthesis via oxidative phosphorylation.^45^ The generation of ATP products of chondrocytes is derived in two ways: one is oxidative phosphorylation and the other is glycolysis.^46^ Notably, chondrocytes reside in a low‐oxygen environment and rely predominantly on glycolysis rather than oxidative phosphorylation (OXPHOS) for energy supply.^13^, ^18^ However, OXPHOS in chondrocytes is significantly more efficient in ATP production compared to glycolysis, yielding 36 ATP molecules per glucose molecule, as opposed to glycolysis's production of only 2 ATP molecules per glucose molecule.^47^, ^48^ ATP products would be reduced with the impaired mitochondrial function.^49^ In this study, we observed distinct effects of varying concentrations of TGF‐β3 on chondrocyte mitochondria. Notably, following a 12‐h exposure, the optimum outcome in terms of mitochondrial abundance and ATP production in chondrocytes was attained at a TGF‐β3 concentration of 5 ng/mL, surpassing other concentration levels. Existing data show TGF‐β appears to stimulate mitochondrial biogenesis and foster energy homeostasis within a moderate concentration, while excessive concentrations of TGF‐β might impose detrimental effects on mitochondrial operation, potentially inducing mitochondrial stress and impairment.^27^, ^50^, ^51^, ^52^, ^53^, ^54^ In the current study, the concentration used was much higher than that on cartilage tissue level (μg/ml vs. pg/ml). In this case, we speculate that TGF‐β3 induced mitochondrial fission and fragmentation might be equivalent to or to or higher than that of the concentration in the knee joint of OA progress. It provided intuitive data support for the understanding of the role of TGF‐β3 in mitochondrial dynamics in OA disease.

As dynamic organelles, mitochondria maintain a state of equilibrium in terms of their number, morphology and function through biogenesis, fusion and fission.^55^, ^56^ Recent studies have highlighted key factors involved in mitochondrial fission in mammalian cells, including Drp1 and Fis1.^18^ Mitochondrial fission helps eliminate damaged mitochondria through a process called mitochondrial phagocytosis.^57^ On the other hand, mitochondrial fusion is regulated by mitochondrial proteins such as Mfn1/2 and Opa1, which control fusion of mitochondria.^19^ Alterations in proteins involved in mitochondrial dynamics have been observed in various conditions,^58^ and these changes in mitochondrial dynamics significantly effect in both physiological and pathological processes in the body.^58^, ^59^ TGF‐β isoforms could also influence mitochondrial dynamics, with TGF‐β1 promoting mitochondrial fission through the phosphorylation of Drp1,^60^ while TGF‐β2 inhibiting mitochondrial networking and promoting mitochondrial fragmentation by suppressing the mitochondrial metabolism‐related protein PGC1.^61^ However, there is limited research on the regulation of mitochondrial dynamics induced by TGF‐β3. This study provided evidence about the role of TGF‐β3 on mitochondrial dynamics in chondrocytes. The findings demonstrated that TGF‐β3 treatment leads to an up‐regulation of mitochondrial fission‐related proteins while down‐regulating mitochondrial outer membrane fusion proteins. These changes in mitochondrial dynamics might further be correlated with inherent mitochondrial cristae morphology.^62^

The AMPKα pathway plays a crucial role in regulating mitochondrial dynamics.^45^ Mitochondria can activate PGC1α through two distinct pathways: the mTOR pathway and the AMPKα pathway, which helps maintain mitochondrial homeostasis by balancing mitochondrial dynamics and energetics.^32^ Phosphorylation of AMPKα is essential for its function, and a decrease in AMPKα phosphorylation inhibits the activity of the NAD^+^‐dependent deacetylase SIRT‐1 and PGC‐1α, which collectively contribute to the regulation of mitochondrial dynamics and energy metabolism, ensuring proper mitochondrial functioning and cellular homeostasis.^60^ In this study, we demonstrated that TGF‐β3 activated the AMPKα signalling, as evidenced by the up‐regulation of AMPKα pathway‐related proteins including p‐AMPKα, AMPKα, SIRT1 and PGC1α (Figure 3A,B). Notably, the expressions of p‐AMPKα and AMPKα showed the most significant increase. These results indicated that TGF‐β3 mediates mitochondrial dynamics in chondrocytes via AMPKα pathway.

We established a correlation between p‐Smad3 signalling and AMPKα signalling in TGF‐β3‐mediated mitochondrial dynamics. By using SIS3, a specific inhibitor for p‐Smad3 signalling, we revealed that SIS3 also blocked the increase of p‐AMPKα, AMPKα, SIRT1 and PCG1 in chondrocytes induced by TGF‐β3. This confirmed that TGF‐β3 activated AMPKα signalling through the p‐Smad3 pathway, thus regulating mitochondrial dynamics in chondrocytes. The TGF‐β/Smads signalling pathway is identified as a canonical pathway involved in this regulation.^55^ However, it is important to note that other signalling pathways, such as the ERK and mTOR pathways, have also been shown to interact with the AMPKα pathway.^23^ Therefore, it is likely that additional pathways contribute to the regulation of mitochondrial dynamics by TGF‐β3 in chondrocytes. Future studies should aim to identify these additional pathways and further elucidate the complex network of signalling events involved in the regulation of mitochondrial dynamics.

**FIGURE 7:**
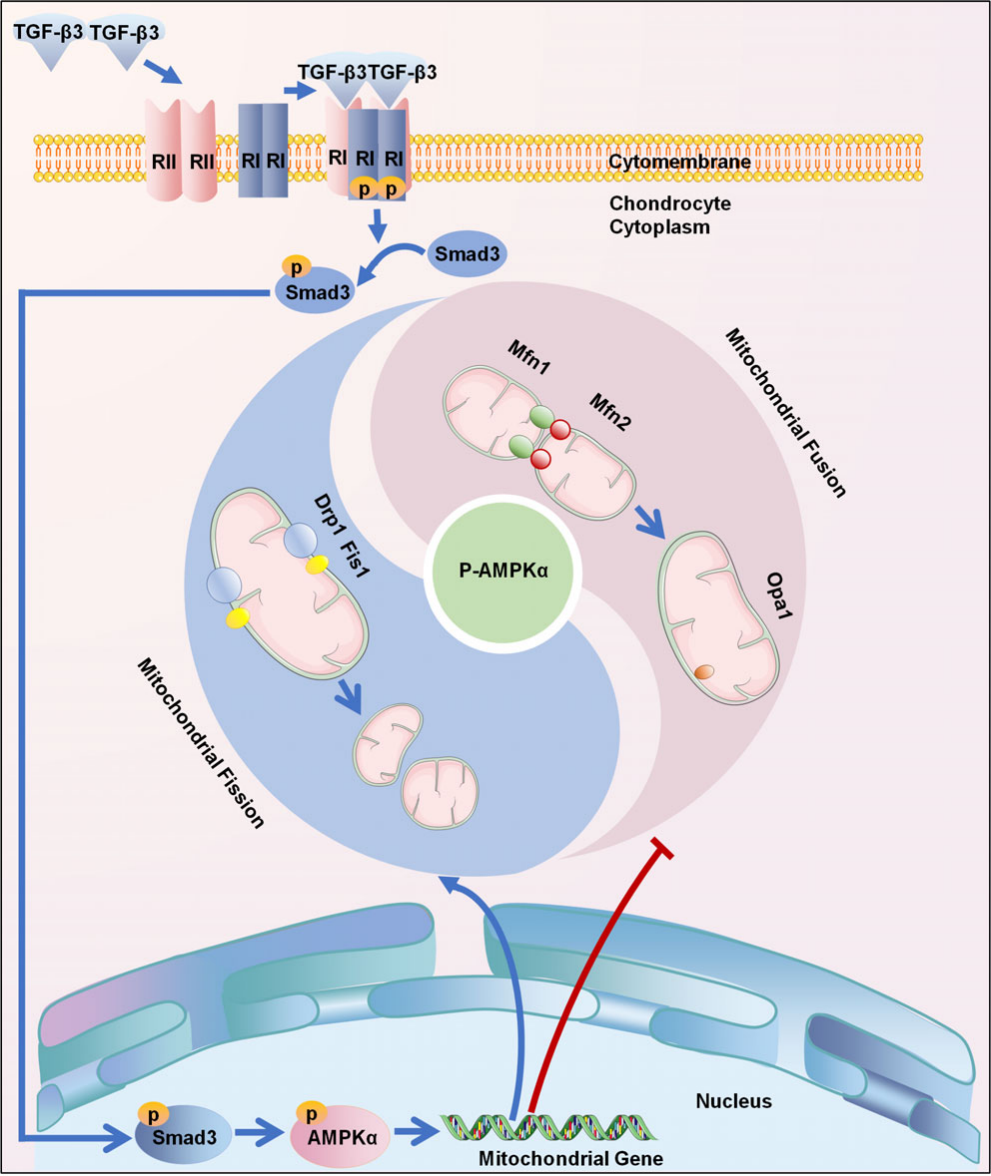
The schematic illustrated the regulatory mechanisms of TGF‐β3 on mitochondrial fusion and fission in chondrocytes. TGF‐β3 binds to RI and RII receptors on chondrocyte membranes to activate the p‐Smad3 pathway. p‐Smad3 pathway further activates AMPK signalling, thereby mediating mitochondria dynamics via mitochondrial fission and fusion in chondrocytes.

In summary, this study investigated the role of TGF‐β3 on chondrocytes mitochondrial dynamics (Figure 7). The results on TGF‐β3‐mediated mitochondrial dynamics indicated the activation of AMPKα signalling and participation of p‐Smad3 signalling. Targeting the TGF‐β3 pathway and modulating mitochondrial dynamics could potentially lead to innovative strategies for the treatment of cartilage‐related conditions.

## FUNDING

We acknowledge financial support from the National Nature Science Foundation of China (81670978 and 81870754 to Xuedong Zhou).

## AUTHOR CONTRIBUTIONS

Jing Xie and Xuedong Zhou designed the experiments. Xinmei Du, Yueyi Yang, Siqun Xu and Jieya Wei performed the experiments and analysed all data. Jiazhou Li and Hao Chen provided suggestions in all experiments and manuscript drafting. All authors reviewed the manuscript, Jing Xie and Xuedong Zhou made a final approval.

## CONFLICT OF INTEREST STATEMENT

The authors report no potential conflicts of interest.

## Supporting information


**Data S1.** Supporting information.

## Data Availability

Derived raw data supporting the results of this study are available from the corresponding author on request.
