# Supplementary material for: TGF‐β3 mediates mitochondrial dynamics through the p‐Smad3/AMPK pathway
Source: Cell Prolif. 2023 Nov 27;57(5):e13579. doi: 10.1111/cpr.13579 (PMC11056712; doi:10.1111/cpr.13579)
Supplement: Supplementary file 1 — Data S1. Supporting information. [file CPR-57-e13579-s001.doc]

Supporting Information File for

*Original article*

**TGF-β3 mediates mitochondrial dynamics through the p-Smad3/AMPK pathway**

Xinmei Du^1^, Mengmeng Duan^1^, Shiyi Kan^1^, Yueyi Yang^1^, Siqun Xu^1^, Jieya Wei^1^, Jiazhou Li^1^, Hao Chen^1^, Xuedong Zhou^1*^, Jing Xie^1*^

1. State Key Laboratory of Oral Diseases & National Center for Stomatology & National Clinical Research Center for Oral Diseases, West China Hospital of Stomatology, Sichuan University, Chengdu 610041, Sichuan, China.

**1. Supplementary Figures**

**Figure S1**


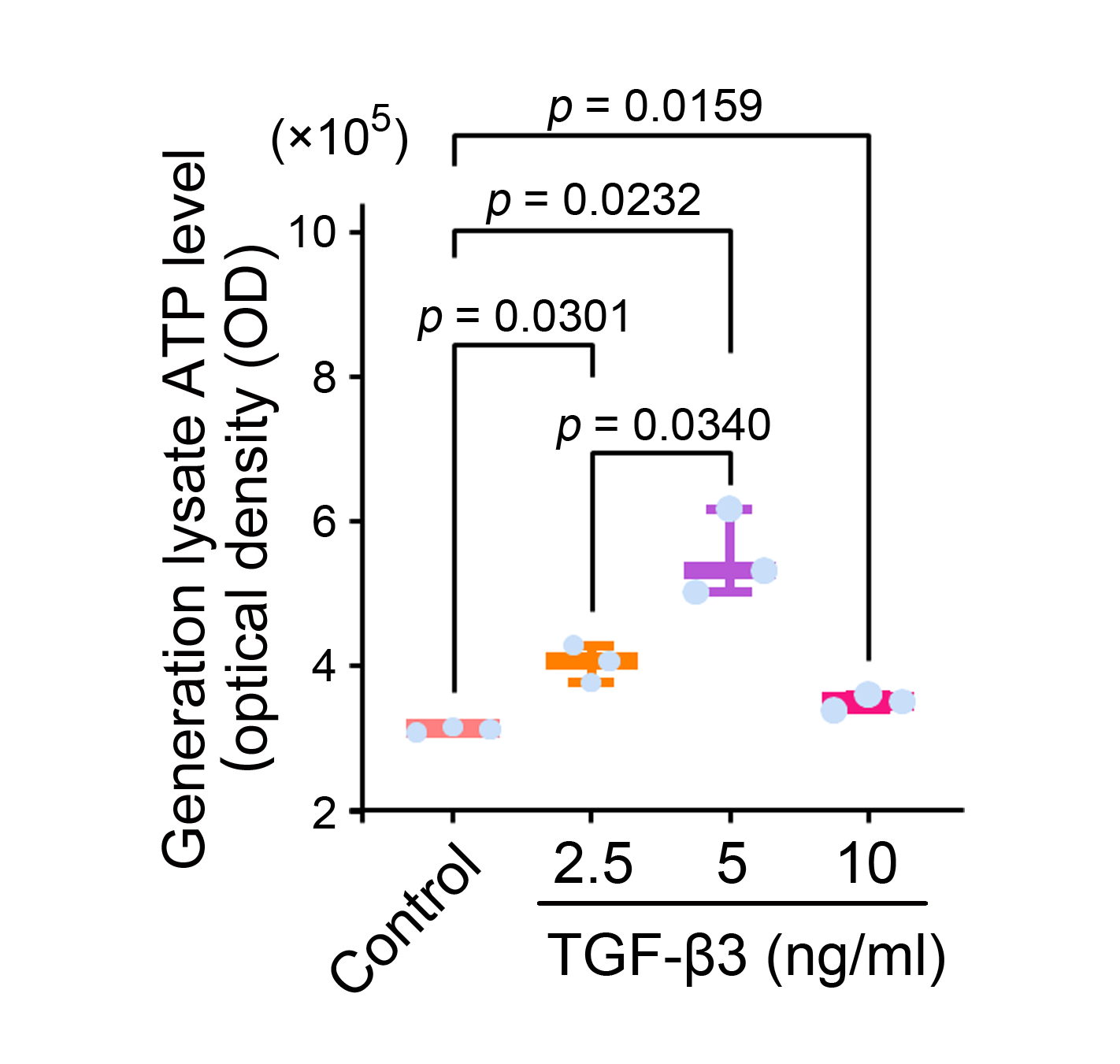


**Figure S1 ATP assay showed the effect of TGF-β3 on ATP products in chondrocytes (n = 3)**. The data were presented as the means ± SD. Student's t-test was applied to determine the significant differences.

**Figure S2**


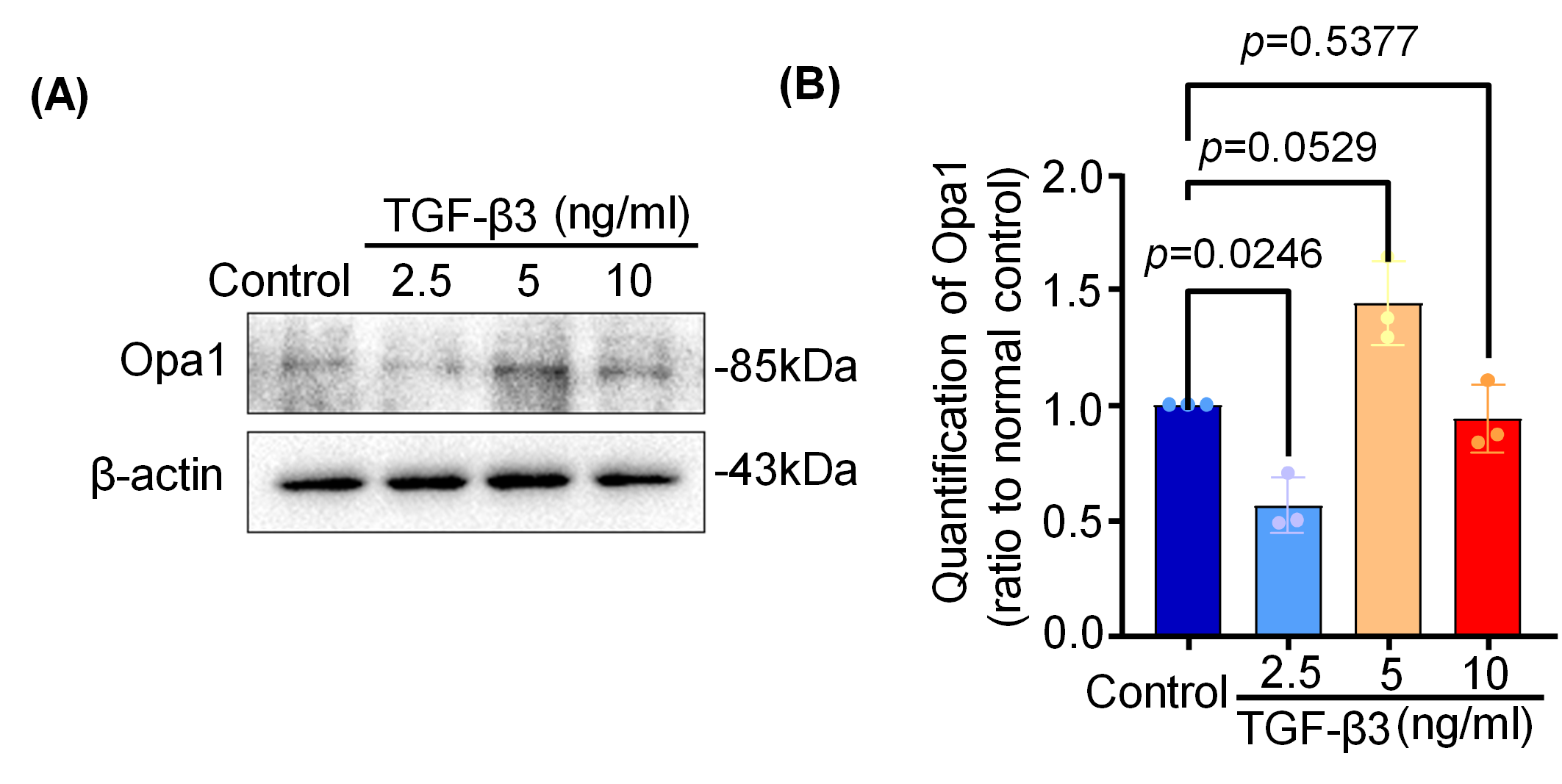


**Figure S2 The expression changes of Opa1 in chondrocytes induced by TGF-β3 at different concentration**. (A) Western blotting showing the expression of Opa1 in chondrocytes induced by TGF-β3 at different concentration (n = 3). (B) Quantitative analysis of Opa1 in chondrocytes induced by TGF-β3 at different concentration (n = 3). The data were shown as box (from 25, 50 to 75%) and whisker (standard deviation, SD). Student's t-test was applied to determine the significant differences.

**2. Supplemental Material-Original (western blotting) data in the study**


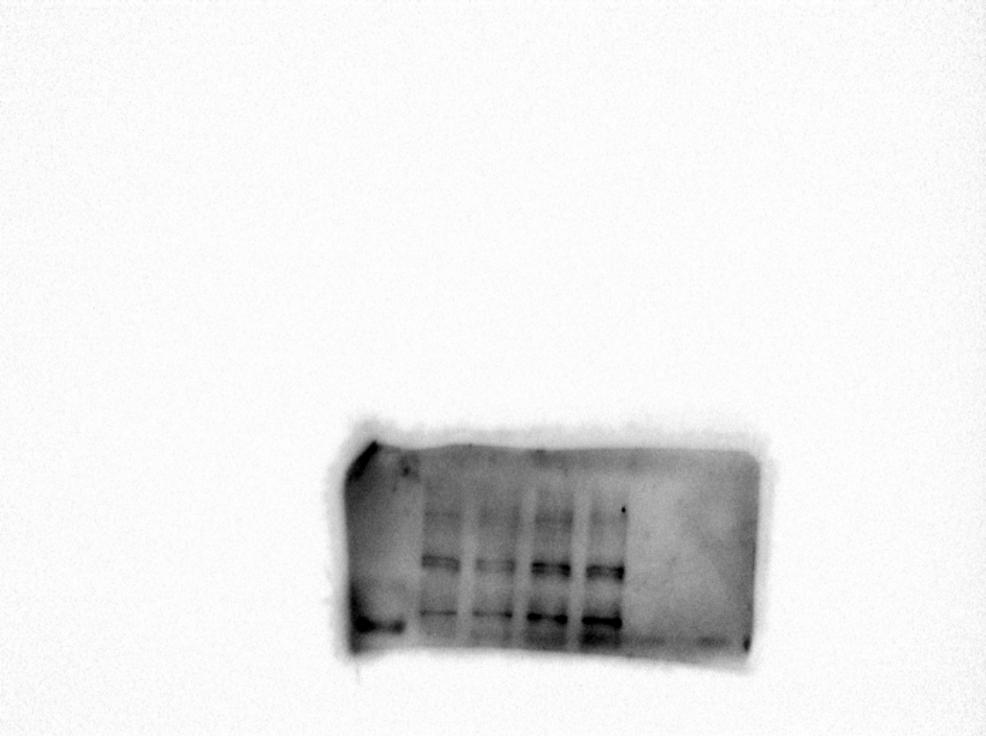
**(1). The original western blots for Figure 2A**

**
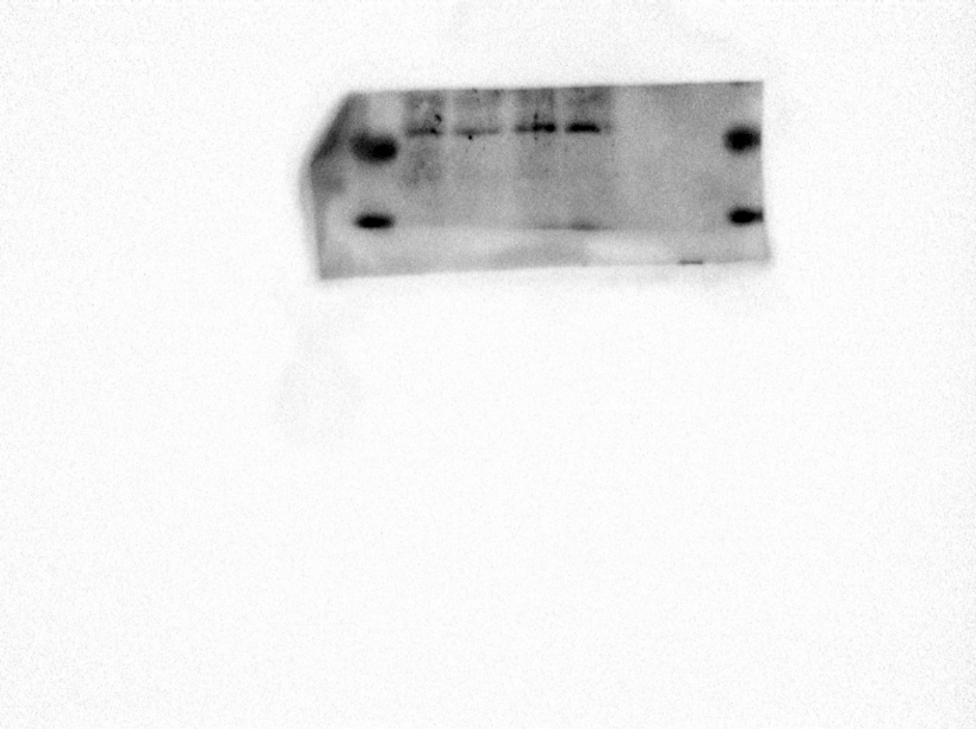
Drp1 Fis1**

**17 kDa**

**82 kDa**

**Fis1**

**Drp1**

**
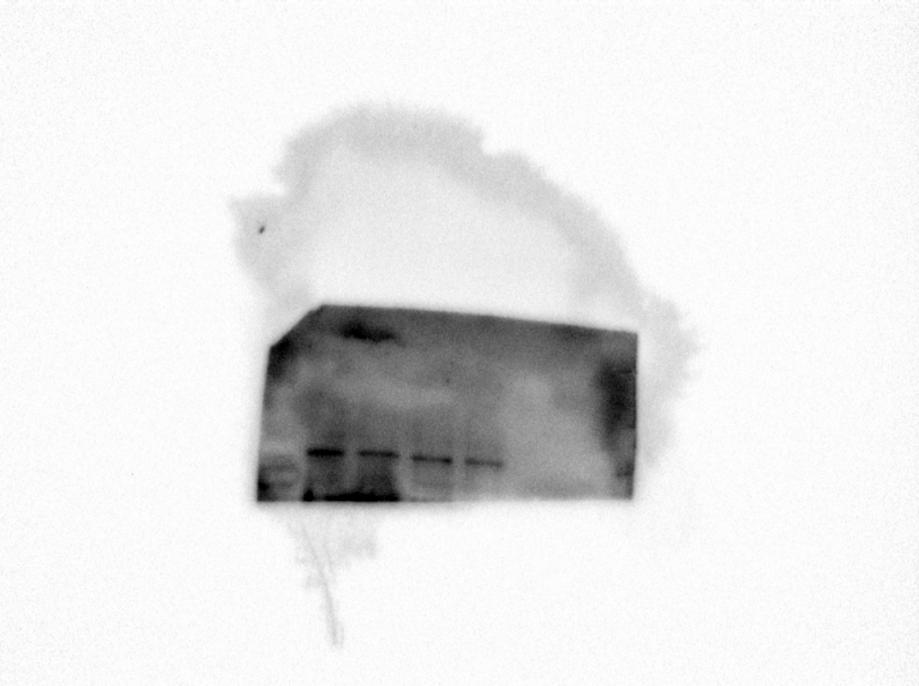
Mfn1
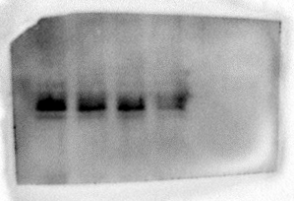
 Mfn2**

**98 kDa**

**86 kDa**

**Mfn1**

**Mfn2**

**
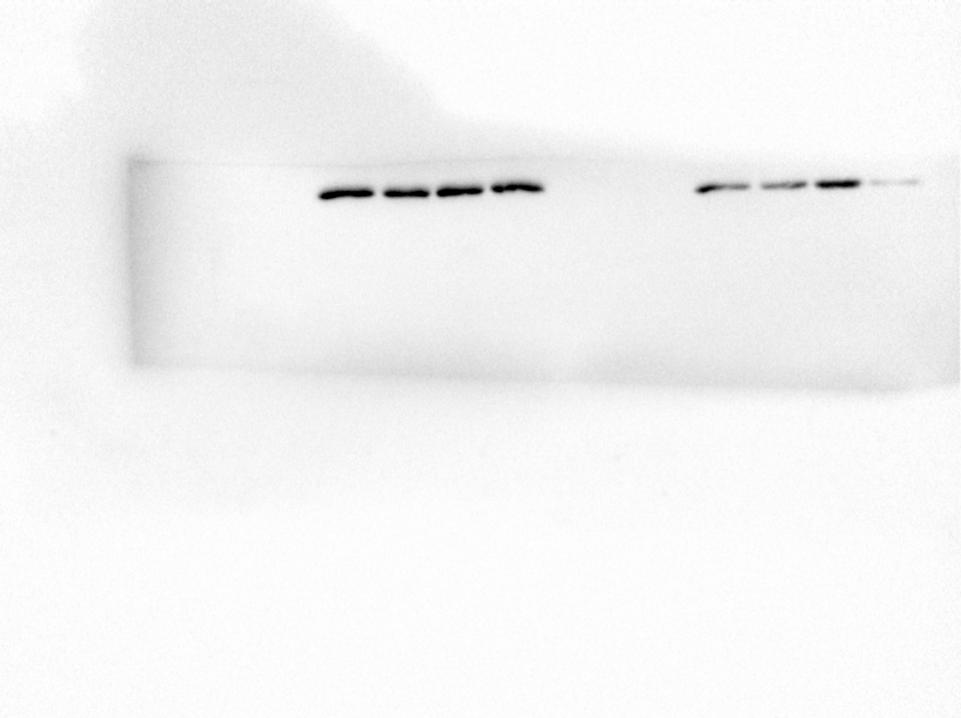
β-actin**

**43 kDa**

**β-actin**


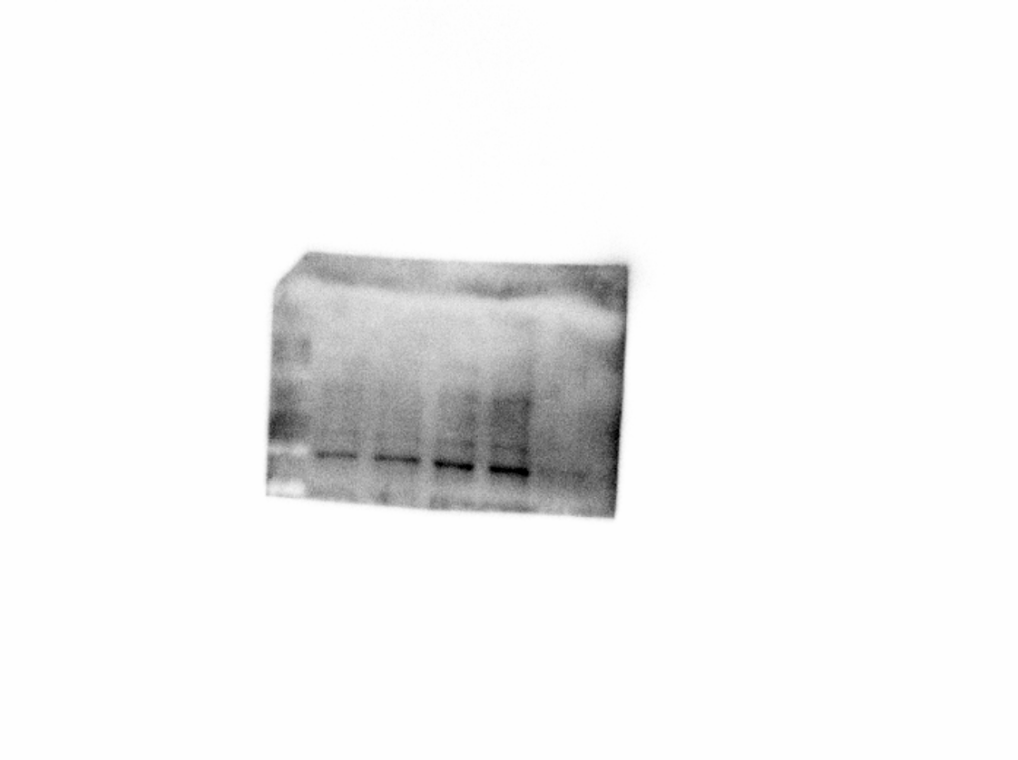
**(2). The original western blots for Figure 4A**


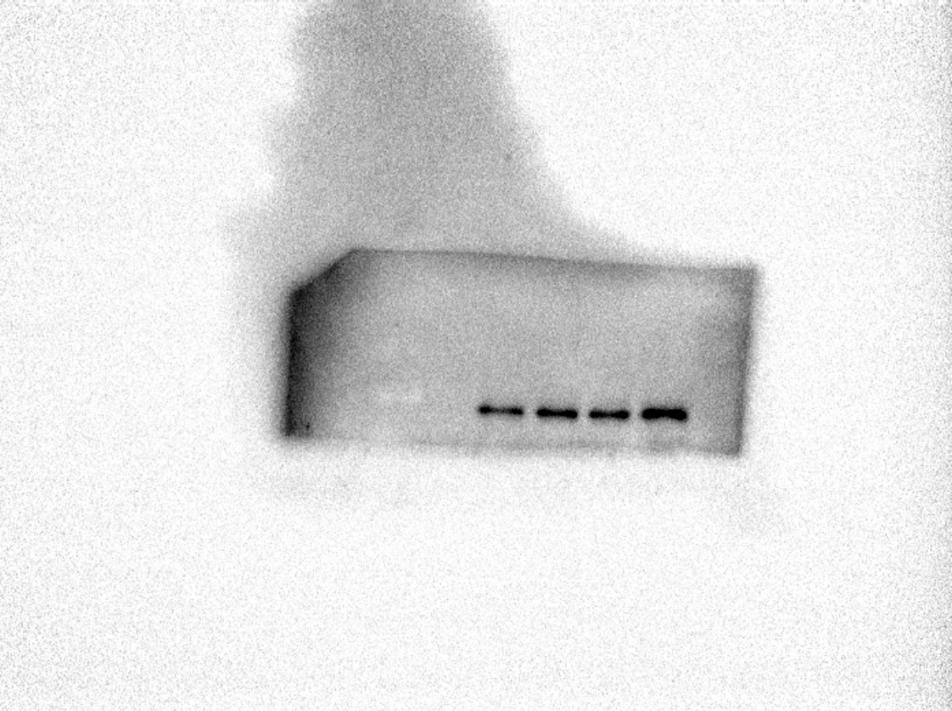
**p-AMPKα AMPKα**

**p-AMPKα**

**120 kDa**

**113 kDa**

**AMPKα**

**
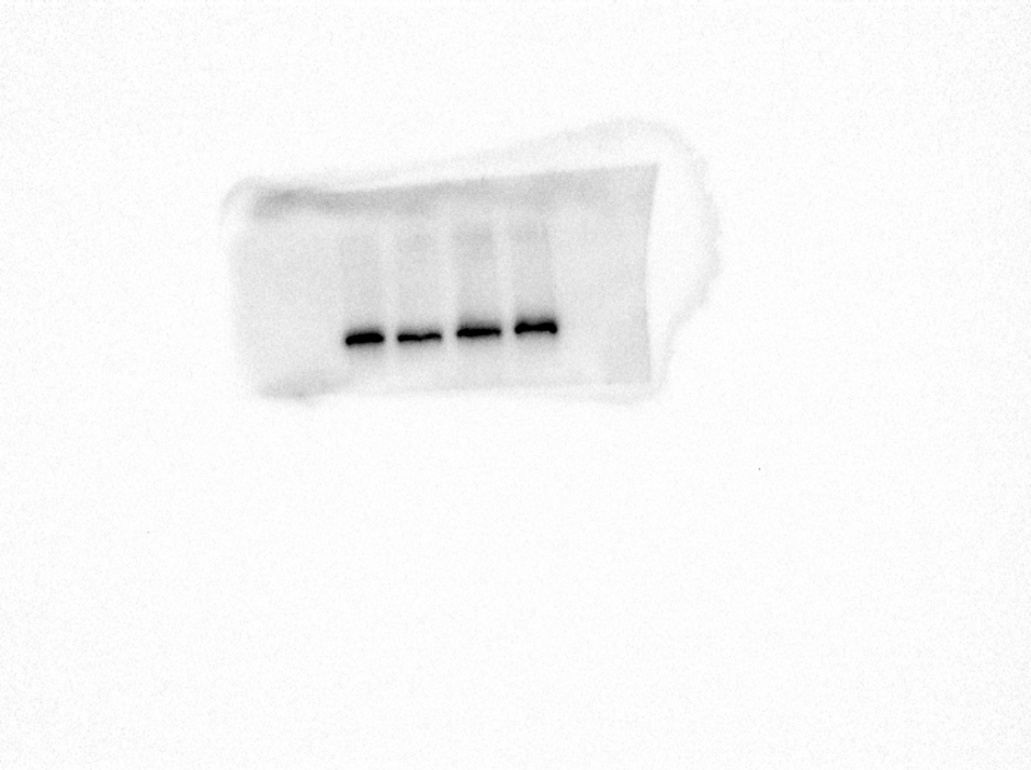

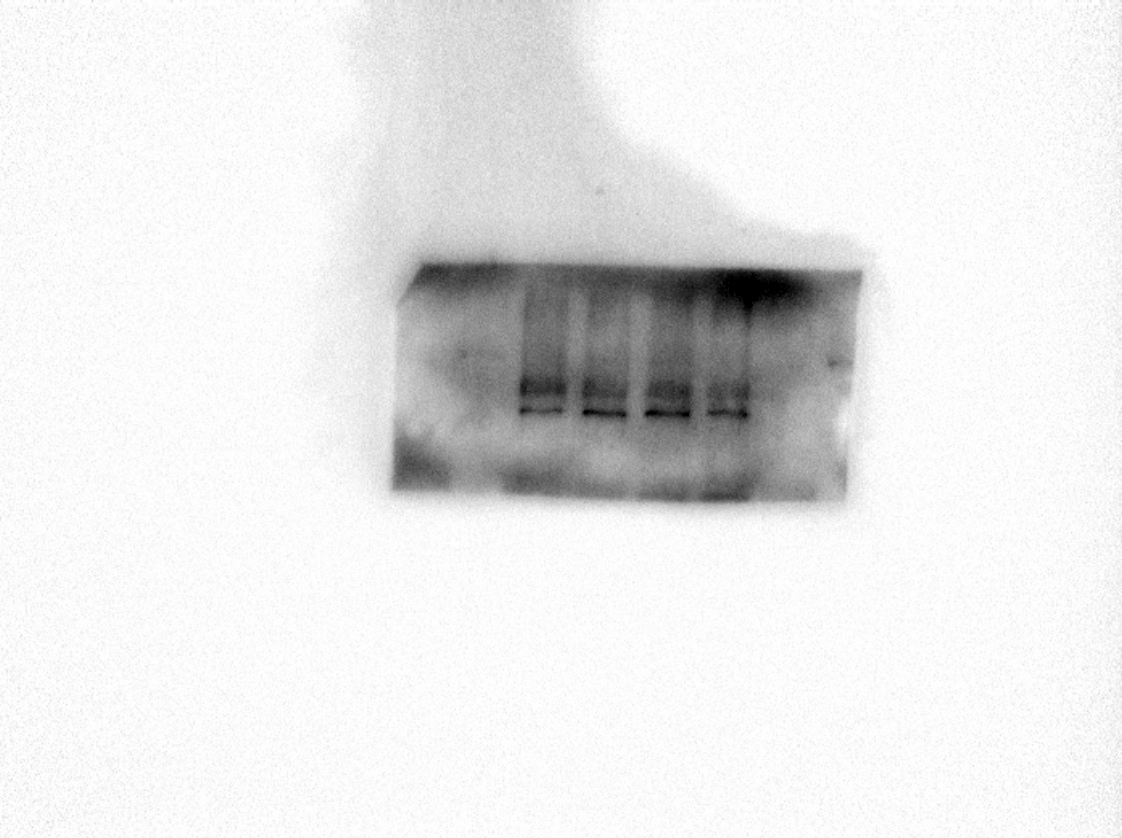
**

**PGC1 SIRT1**

**PGC1**

**SIRT1**

**62 kDa**

**62 kDa**

**
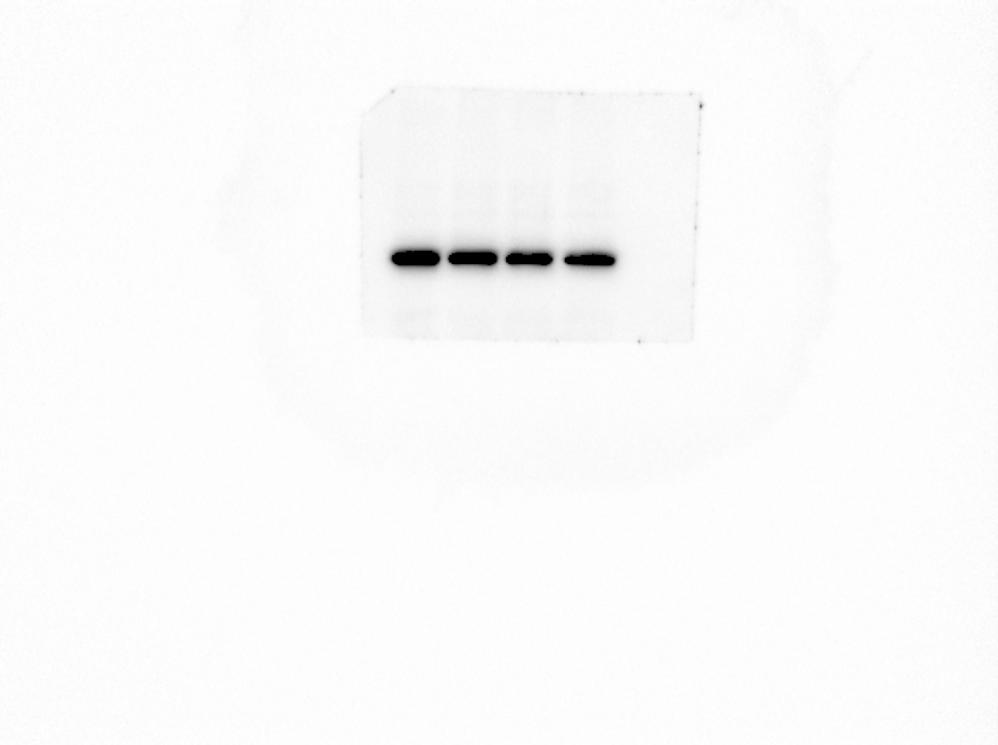
β-actin**

**β-actin**

**43 kDa**


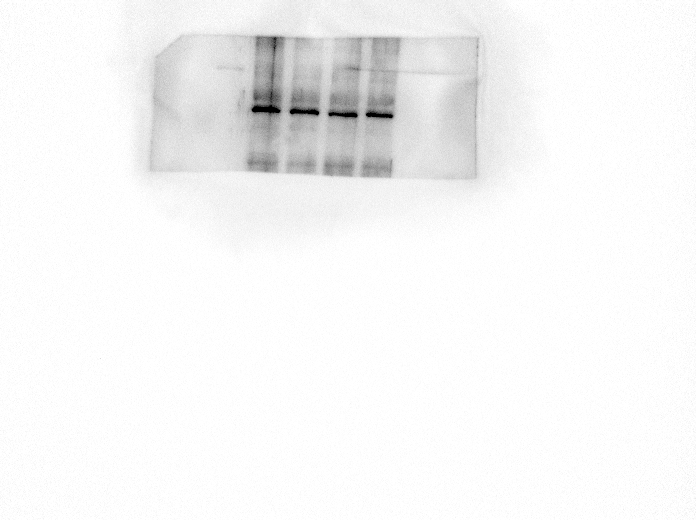
**(3). The original western blots for Figure 5A**


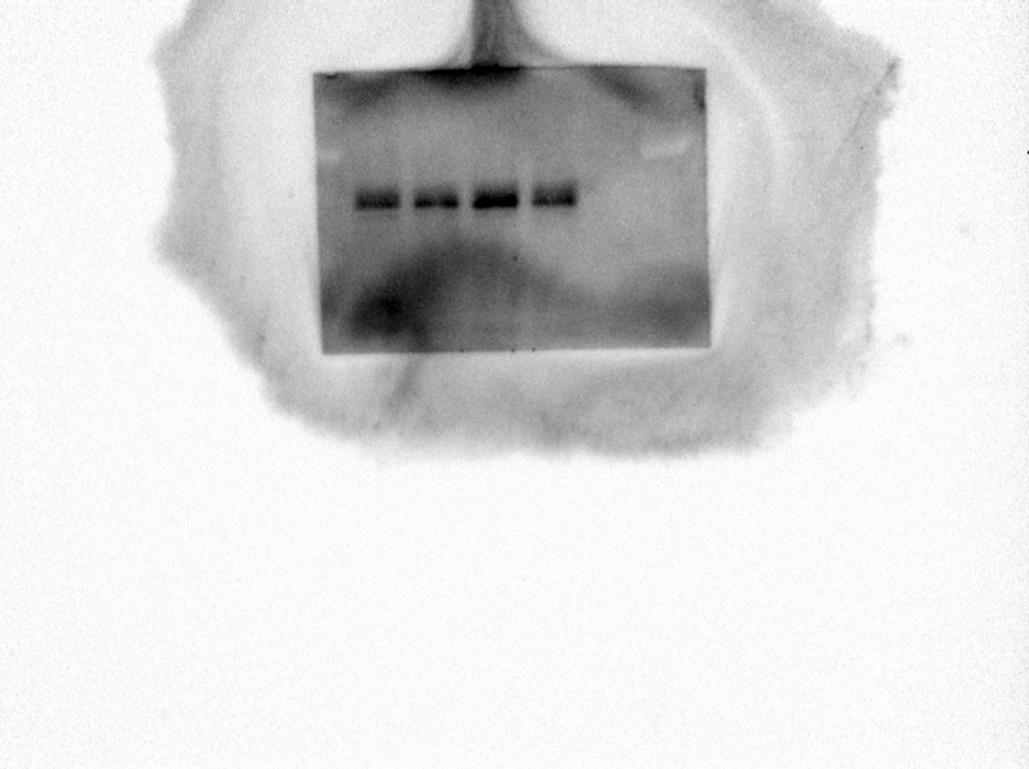
**P-Smad3 Smad3**

**p-Smad3**

**58 kDa**

**58 kDa**

**Smad3**


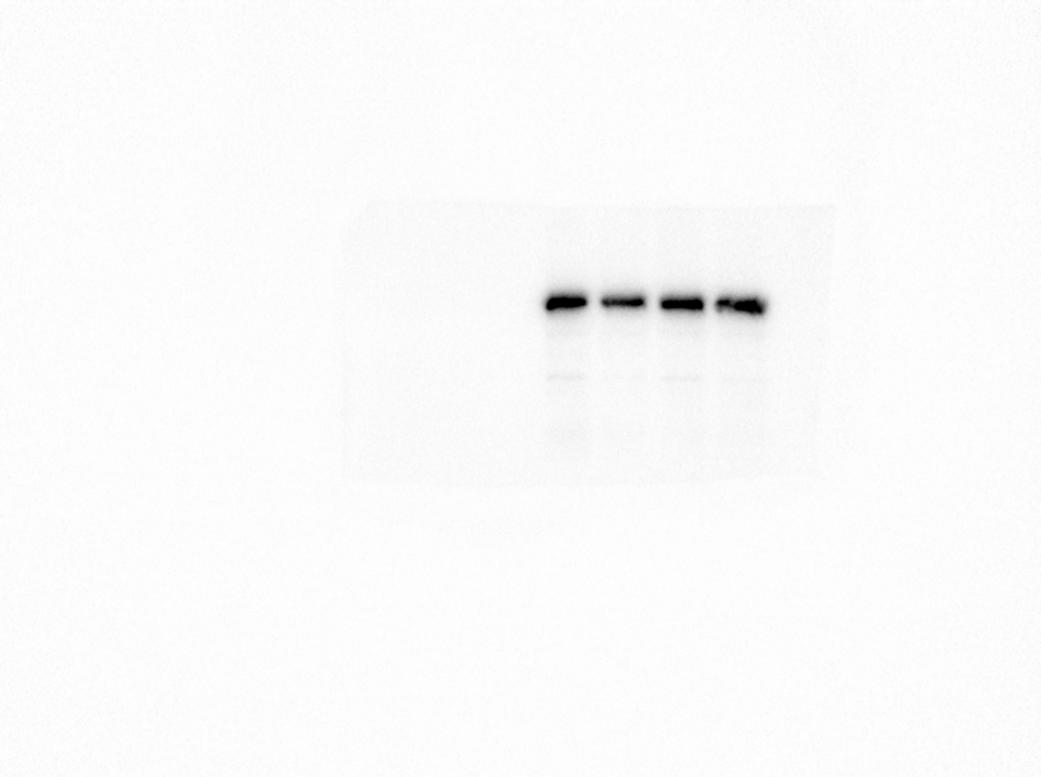
**Smad4 β-actin**

**
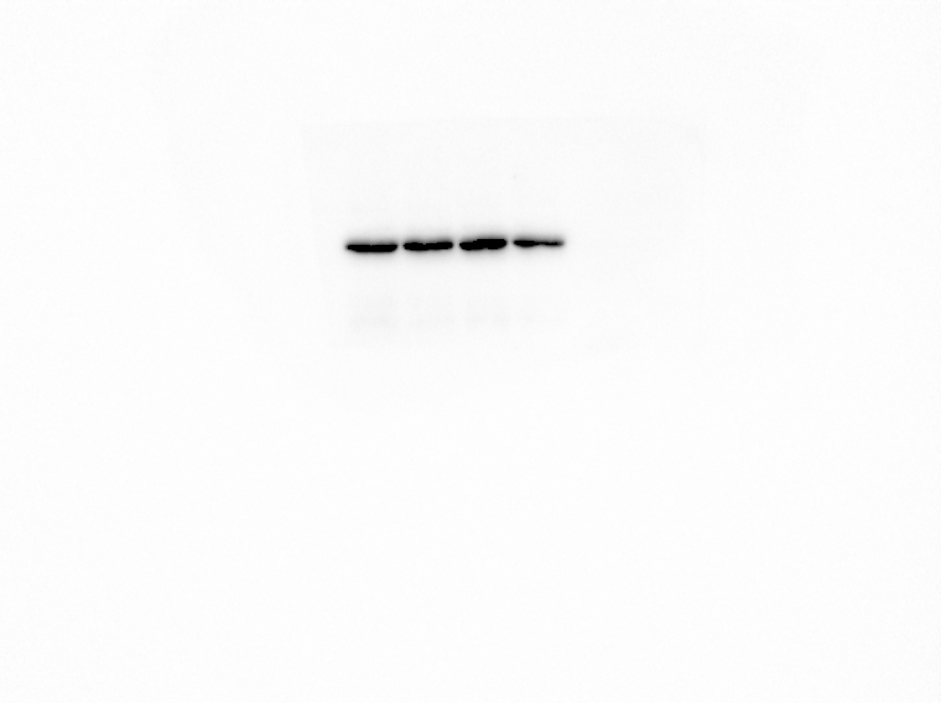
**

**Smad4**

**60 kDa**

**β-actin**

**43 kDa**

**(4). The original western blots for Figure 6A**


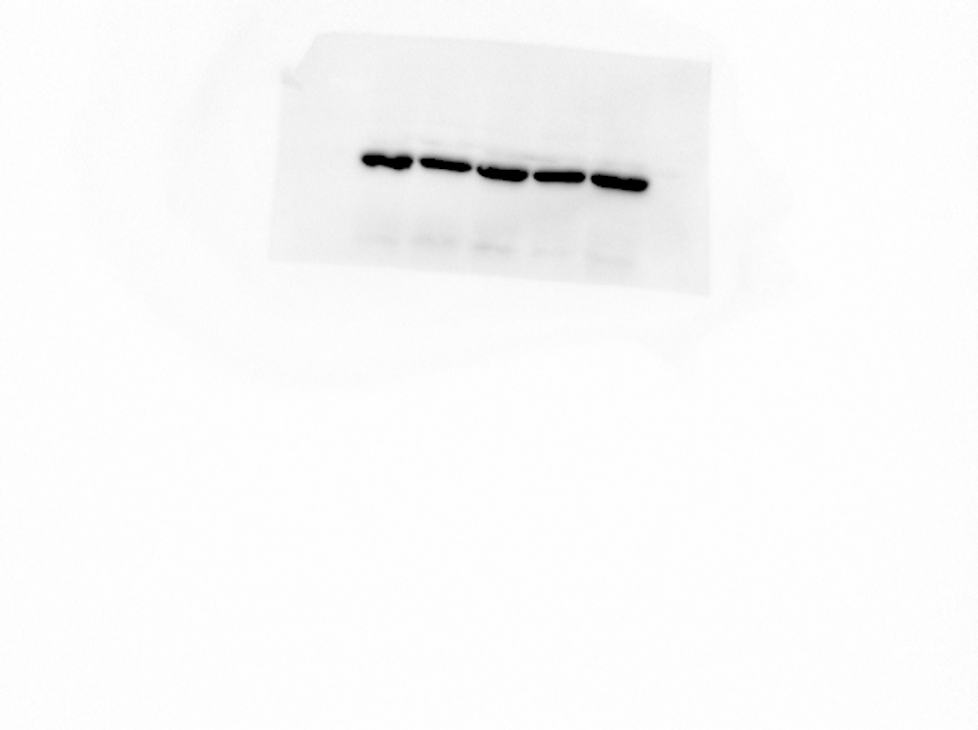
**p-Smad3 β-actin**

**
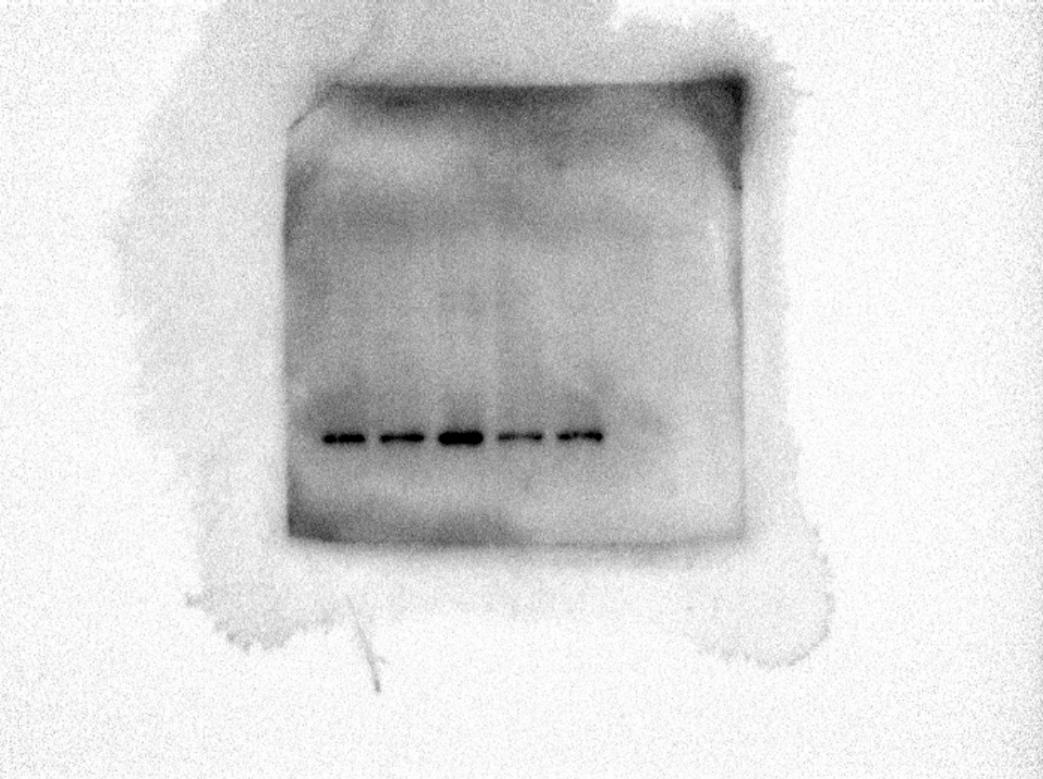
**

**β-actin**

**43 kDa**

**P-Smad3**

**58 kDa**

**(5). The original western blots for Figure 6D**

**
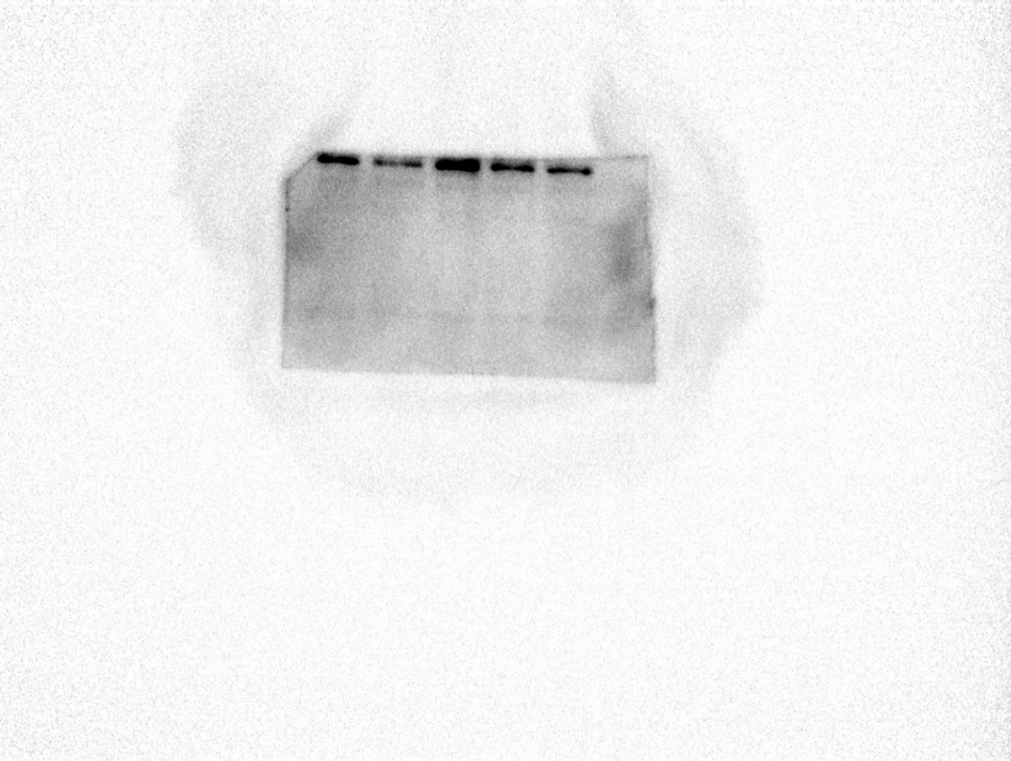
p-AMPKα AMPK-α**

**AMPKα**

**62 kDa**

**
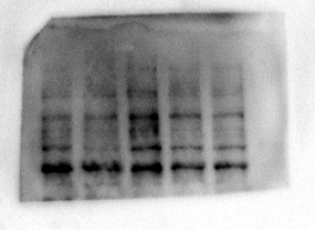
**

**p-AMPKα**

**62 kDa**

**
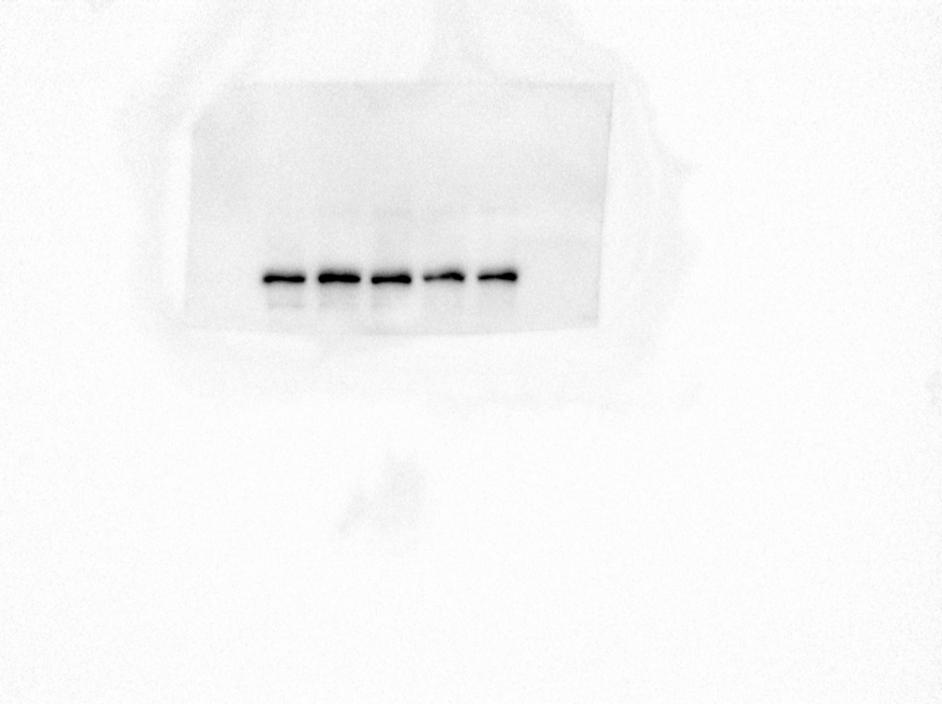
**


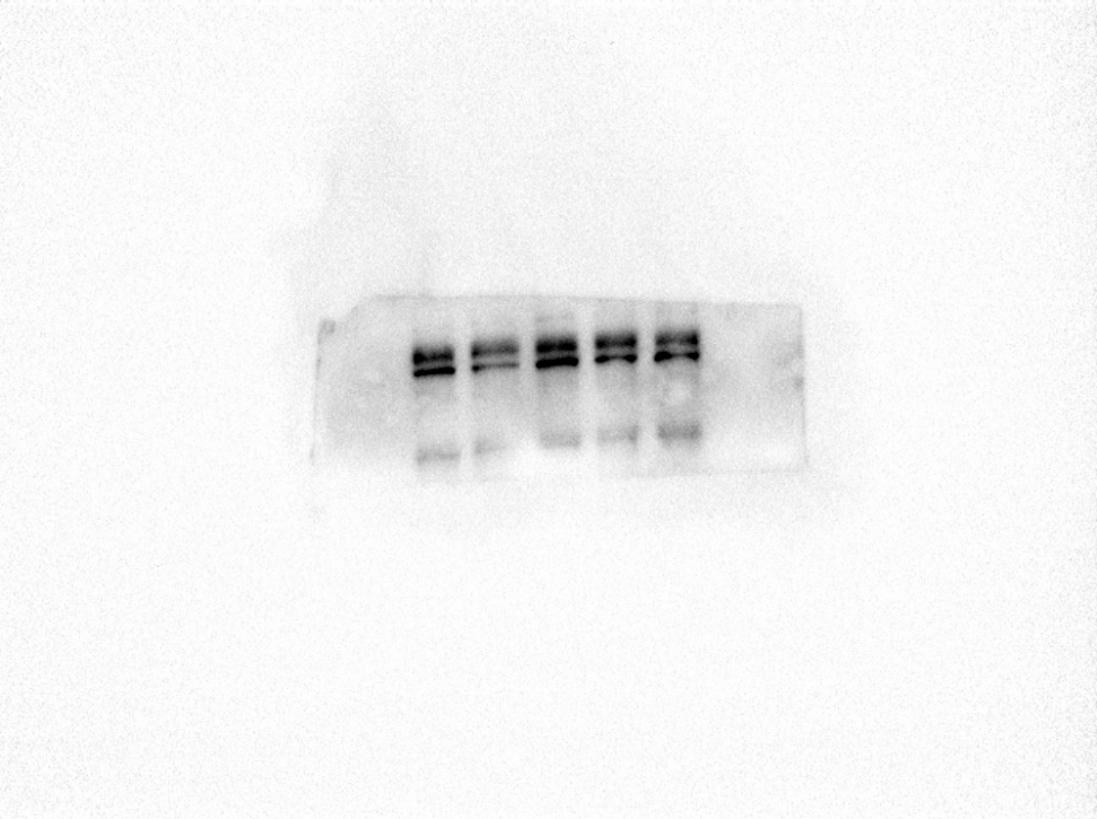
**PGC1 SIRT1**

**PGC1**

**113 kDa**

**SIRT1**

**120 kDa**

**β-actin**

**
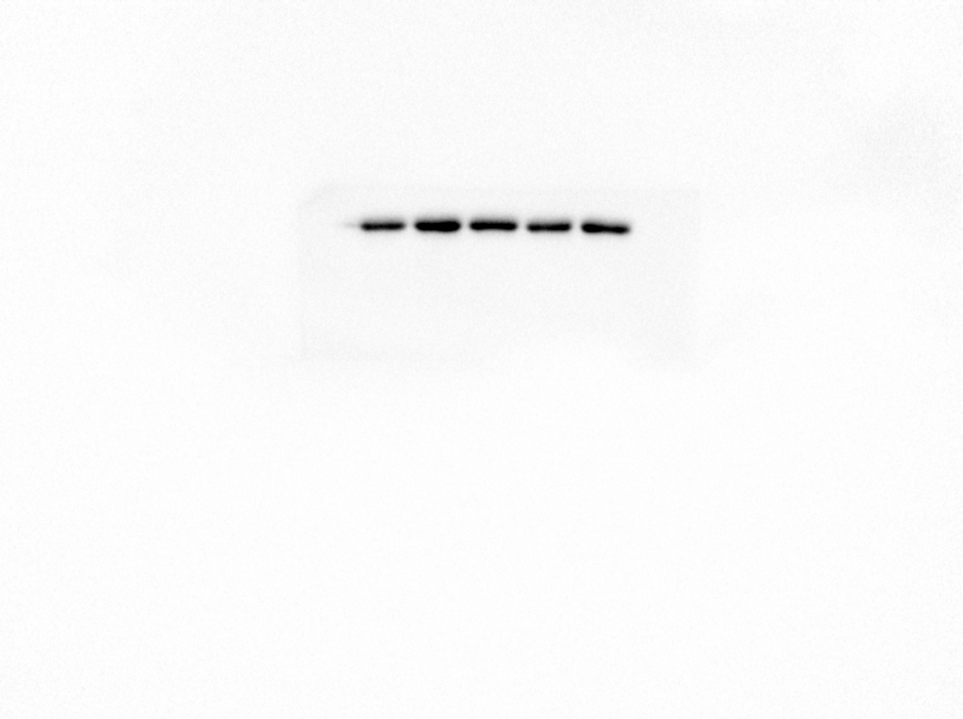
**

**β-actin**

**43 kDa**

**(6).The original western blots for Figure 6G**


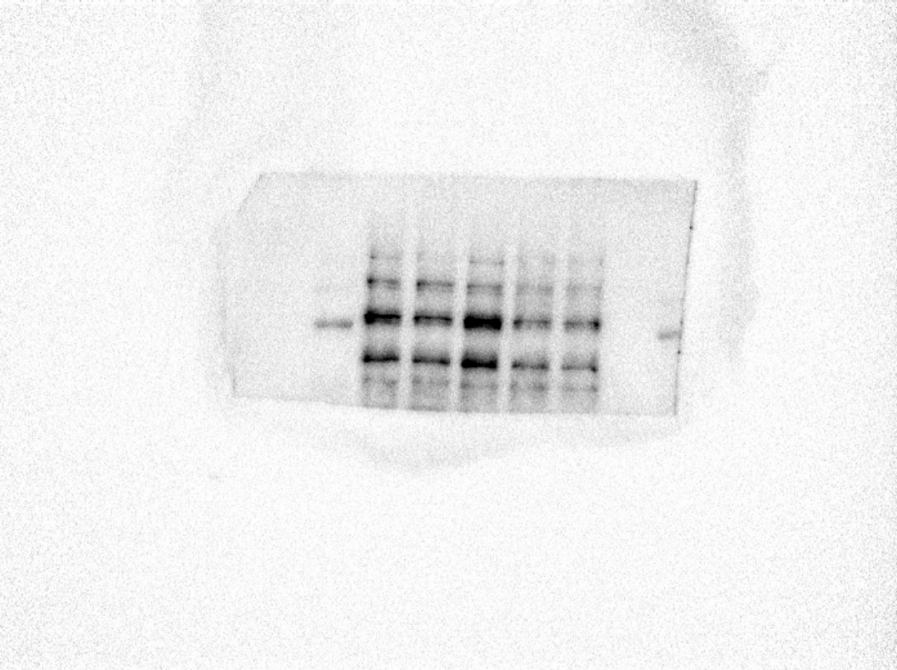
**Drp1 Fis1**

**
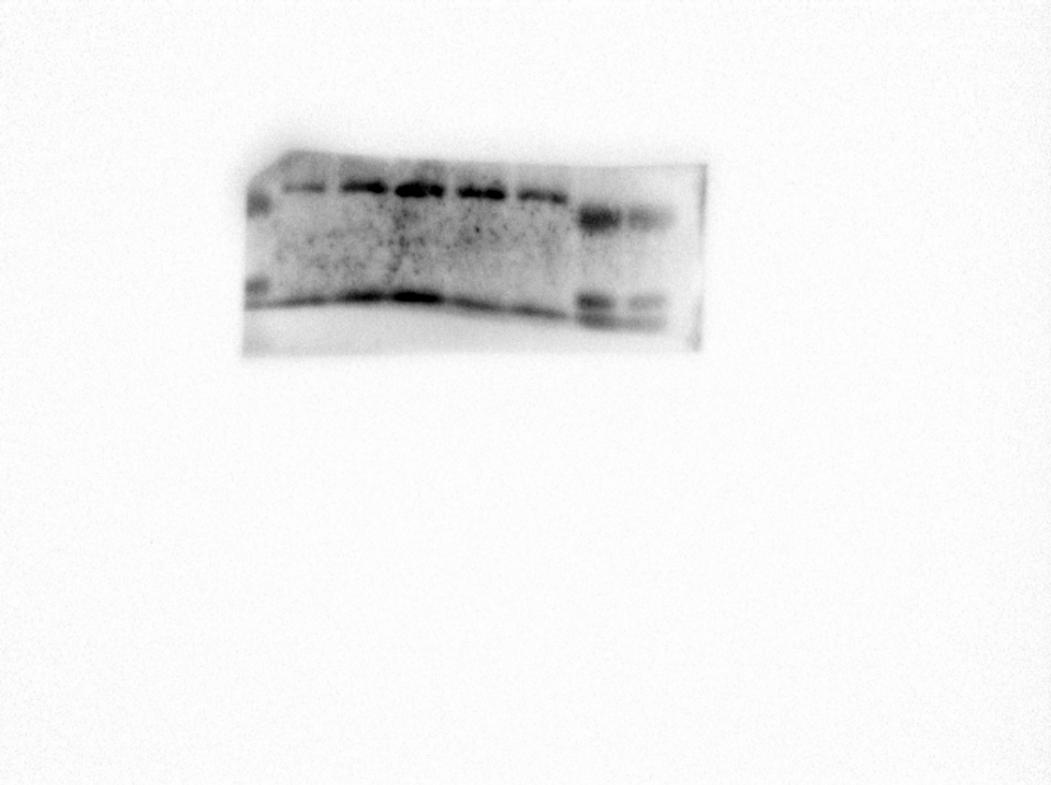
**

**Drp1**

**Fis1**

**17 kDa**

**82 kDa**


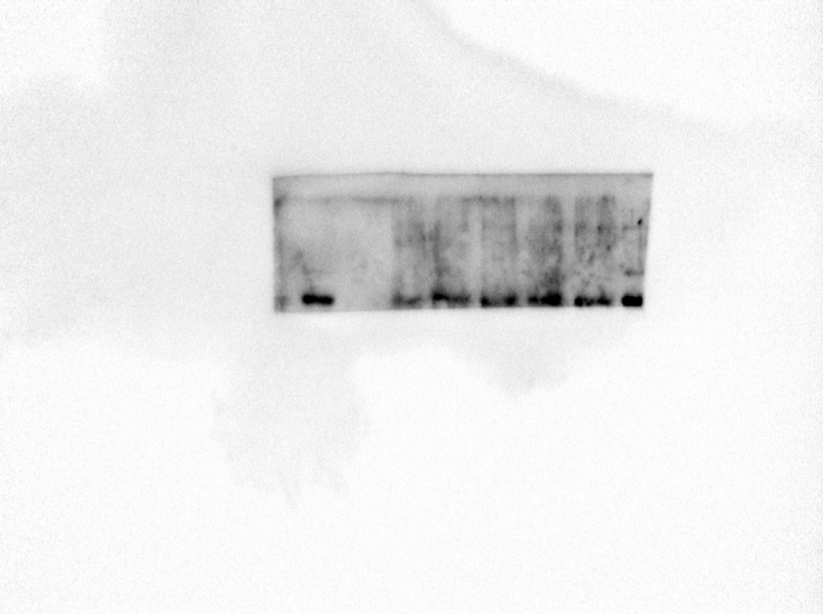
**Mfn1 Mfn2**

**
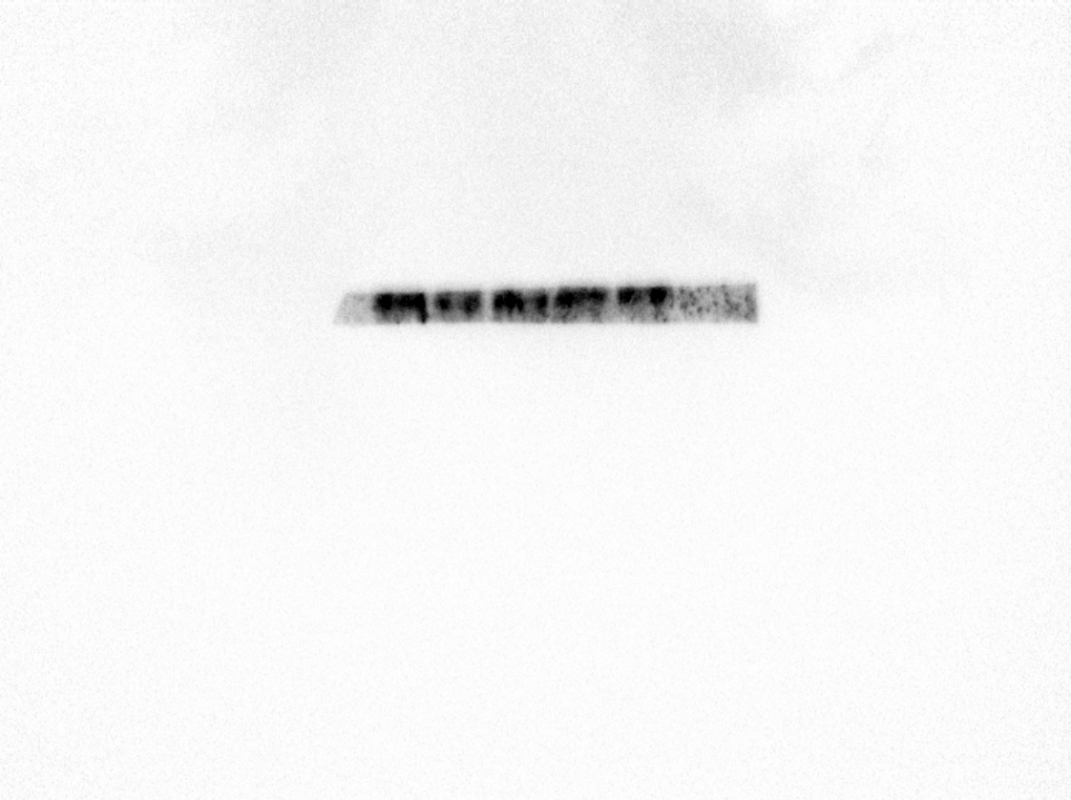
**

**Mfn1**

**86 kDa**

**Mfn2**

**98 kDa**


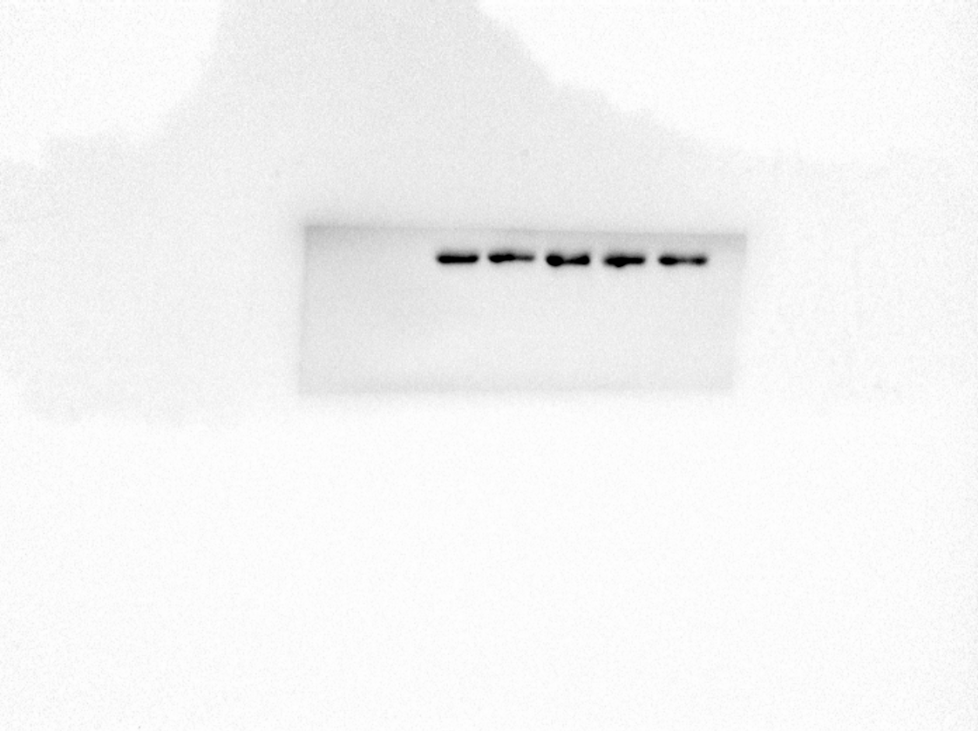
**β-actin**

**β-actin**

**43 kDa**

**(7).The original western blots for Figure S2A**

**
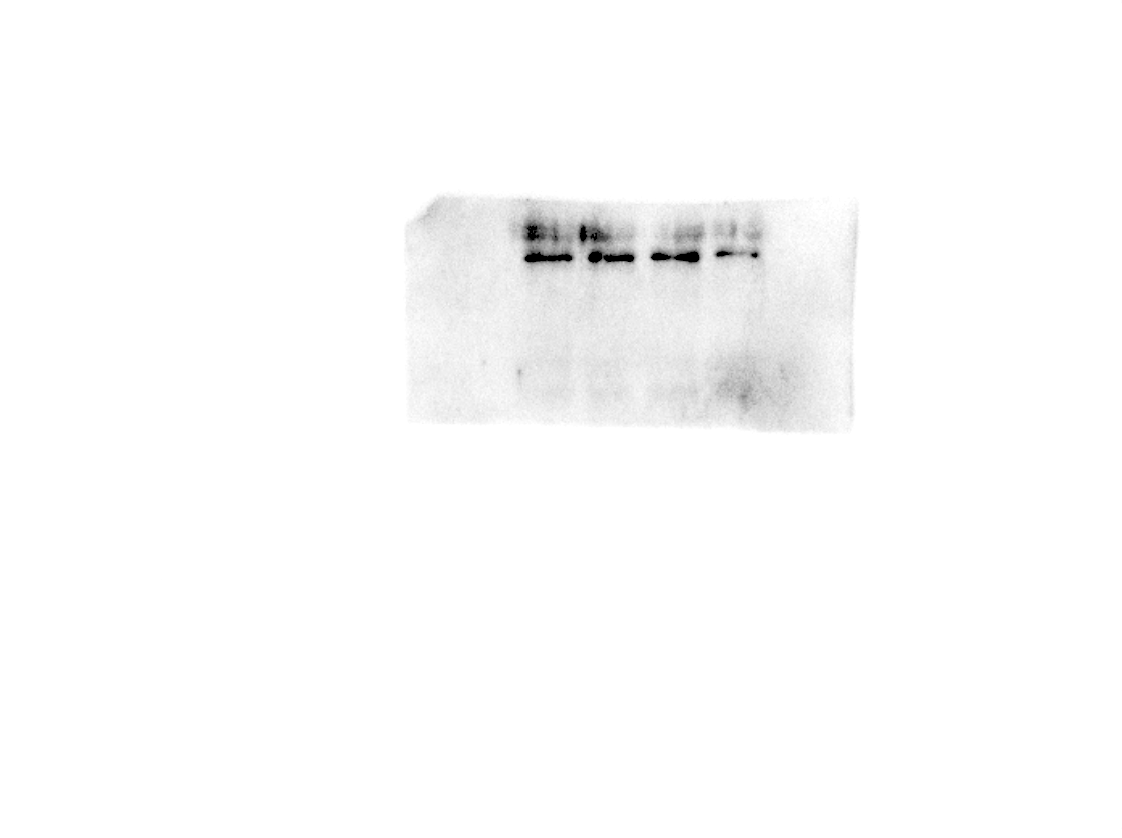
Opa1**

**Opa1**

**85 kDa**
